# Spinal cord stimulation to manage autonomic dysfunction after spinal cord injury: a systematic review

**DOI:** 10.3389/fnhum.2026.1763475

**Published:** 2026-06-03

**Authors:** Pratham Upadhyay, Ioannis Tsikopoulos, Clement Hiscock, Hannah Houliston, Sean Doherty, Sarah Knight, David Baxter, Lynsey Duffell

**Affiliations:** 1 Department of Medical Physics and Biomedical Engineering, University College London, London, United Kingdom; 2 London Spinal Cord Injury Centre, Royal National Orthopaedic Hospital, London, United Kingdom; 3 Department of Spinal Surgery, The Whittington Hospital, London, United Kingdom

**Keywords:** autonomic function, bladder, bowel, cardiovascular, sacral anterior root stimulation, sexual function, spinal cord injury, spinal cord stimulation

## Abstract

**Introduction:**

Spinal cord injury (SCI) results in severe autonomic dysfunction, across cardiovascular, bladder, bowel and sexual domains, negatively affecting quality of life. Recently, there has been growing interest in the application of spinal cord stimulation (SCS) to restore or modulate autonomic functions in people with SCI. This review systematically evaluated the evidence for spinal cord and/or root stimulation, delivered via epidural (eSCS), transcutaneous (tSCS) spinal cord stimulation, or sacral (posterior) anterior root stimulation (SARS/SPARS) – in managing autonomic dysfunction after SCI.

**Methods:**

A systematic search of OVID Medline, OVID Embase, and Web of Science, was completed in September 2024. Eligible studies included adults with SCI (≥18 years old) receiving eSCS, tSCS, or SARS, reporting at least one quantitative autonomic outcome measure. Extracted data were synthesised by autonomic domain (cardiovascular, urinary, bowel, sexual), intervention type (eSCS, tSCS, or SARS) and duration of stimulation (acute vs. chronic).

**Results:**

Fifty-seven articles were identified, evaluating eSCS (30 studies, *n* = 151), tSCS (10 studies, *n* = 48), and SARS (17 studies, *n* = 1,249) effects on cardiovascular (22 studies), bladder (23 studies), bowel (14 studies), and sexual function (13 studies). Acute application of eSCS or tSCS consistently elevated blood pressure and mitigated orthostatic hypotension, with variable effects on suppressing autonomic dysreflexia. Acute application of eSCS, tSCS or SPARS, improved bladder capacity and voiding efficiency, although not consistently and often outcomes did not reach clinically acceptable levels. eSCS, tSCS and SARS consistently reduced bowel management times. In most participants, erections could be achieved or were improved with eSCS or SARS. Chronic interventions, particularly SARS with dorsal root deafferentation, produced long term bladder and bowel improvements, whereas chronic eSCS and tSCS evidence was limited. Stimulation was most often applied to lumbosacral segments (L1–S1) at 30–60 Hz, although some studies used systematic mapping approaches.

**Conclusion:**

Electrical stimulation of the spinal cord and/or nerve roots is a promising method for modulating or restoring autonomic function after SCI. However, current evidence is based on small cohort or case studies, or retrospective reporting. Larger controlled studies are recommended to establish efficacy and long-term safety, and further mechanistic investigations are required.

**Systematic review registration:**

https://www.crd.york.ac.uk/PROSPERO/view/CRD420250621447, identifier PROSPERO (CRD420250621447).

## 1 Introduction

There are approximately 105,000 people living with Spinal Cord Injury (SCI) in the UK. With around 4,000 new cases every year and average lifetime costs of £1.1 m (McDaid et al., 2019). Spinal cord injuries are life-changing events resulting in the partial or complete loss of sensory and motor function. Other, less visible effects of SCI include severe autonomic dysfunction manifesting as cardiovascular, bladder, bowel, sexual and thermoregulatory dysfunctions. These severely impact quality of life, increasing morbidity and mortality, and remain key priorities amongst the SCI population (Anderson, 2004; van Middendorp et al., 2016). Neurological control of autonomic function is complex, involving a range of spinal sympathetic and parasympathetic networks under supraspinal regulation. The level of autonomic dysfunction present depends on the neurological level and severity of the SCI.

Cardiovascular function, can be severely compromised after SCI. People with SCI, specifically at levels above T6, are prone to haemodynamic instability, leading to both significant decreases (orthostatic hypotension (OH)), as well as increases (autonomic dysreflexia (AD)) in blood pressure (BP), due to impairments in sympathoexcitation and sympathoinhibition, respectively. These states of haemodynamic instability can lead to debilitating symptoms such as light-headedness, dizziness, blurred vision in hypotensive states, and sweating, flushing and headaches during onset of AD. Furthermore, in the long term these cardiovascular changes have been shown to increase the incidence of cerebrovascular and heart disease (Phillips and Krassioukov, 2015).

Bladder, bowel and sexual dysfunction affect almost all individuals with SCI. Up to 95% of individuals with supra-sacral SCI have bladder overactivity (or Neurogenic Detrusor Overactivity (NDO)), often associated with loss of co-ordination between the detrusor muscle and urethral sphincters (Detrusor Sphincter Dyssynergia (DSD)), leading to incontinence, impaired bladder emptying, urinary tract infections (UTIs), and long-term renal damage. Management after SCI typically relies on catheterisation and pharmacological agents, both associated with burdensome side effects. Nonetheless, UTIs and incontinence remain highly prevalent (Ali and Jacques, 2019).

Chronic bowel dysfunction is also common (Myers et al., 2019). Constipation affects ~ 80% of people (Feng et al., 2025) with over half experiencing faecal incontinence (Coggrave et al., 2014; Sober-Williams et al., 2024). Gut transit can be 2–4 times slower in individuals with SCI (Willits et al., 2024; Glickman and Kamm, 1996), contributing to constipation, abdominal bloating (Feng et al., 2025) and subsequent complications such as haemorrhoids, anal fissures, faecal impaction and pelvic organ prolapse. Daily bowel management routines often last 1–2 h and commonly prescribed medications after SCI (anticholinergics and antispasmodics) can also further impact bowel function.

Sexual dysfunction is also prevalent after suprasacral SCI. Although sacral spinal reflex pathways remain intact, allowing reflexogenic erections in men and lubrication in women—these responses are too short-lived or unreliable (Krassioukov and Elliott, 2017). Particularly, impaired sensory function and loss of supraspinal input, impair psychogenic arousal and orgasm, limiting sexual function and satisfaction. Pharmacological agents (e.g., PDE-5 inhibitors) or Vacuum Pump devices can support erectile function, but do not fully address the scope of sexual dysfunction in SCI. Uninhibited reflexogenic erections can be problematic, and impaired coordination of the urethral sphincters can cause retrograde ejaculation.

Over many decades, electrical stimulation (ES) has been used in SCI rehabilitation with well-established benefits to musculoskeletal and autonomic health. Our group and others have shown that invasive (e.g., Sacral Anterior Roots Stimulation (SARS)) or non-invasive (e.g., Dorsal Genital Nerve Stimulation (DGNS)) ES applied over nerve roots or peripheral nerves can improve continence (Hu et al., 2019; Doherty et al., 2020), reduce bladder overactivity (Kirkham et al., 2001; Kirkham et al., 2002; Doherty et al., 2020), increase bladder capacity (Kirkham et al., 2001; Kirkham et al., 2002; Bourbeau et al., 2018; Doherty et al., 2020), and restore bladder and bowel emptying (Brindley et al., 1986) in people with SCI. Despite these promising results, pudendal nerve stimulation has drawbacks, limiting its uptake. Additionally, only one or two nerve roots (S2–S3) are engaged, whereas innervation of autonomic function spans multiple spinal levels.

Spinal cord stimulation (SCS) involves placing electrodes over the spinal cord, either transcutaneously or within the epidural space, simultaneously stimulating multiple posterior nerve roots (Murg et al., 2000; Rattay et al., 2000; Minassian et al., 2007; Ladenbauer et al., 2010). SCS has been used for decades in the treatment of chronic neuropathic pain, and, over the past 20 years, several groups have reported its potential for restoring a degree of motor control within chronic SCI (Harkema et al., 2011; Hofstoetter et al., 2015; Angeli et al., 2018; Darrow et al., 2019; Al'joboori et al., 2020). More recently, there has also been growing interest in the potential benefits of SCS for autonomic dysfunction after SCI, due, in part, to anecdotal reports of improved bladder, bowel and cardiovascular function in people undergoing SCS for lower limb motor control (Harkema et al., 2011; Grahn et al., 2017; Darrow et al., 2019; Herrity et al., 2021). SCS preferentially recruits large-diameter dorsal root afferents (Ladenbauer et al., 2010), possibly modulating spinal interneuronal networks (Schramm, 2006), and altering output of overlapping sensorimotor and autonomic circuits (Samejima et al., 2023b). Some groups have specifically attempted to target autonomic functions via epidural (eSCS) (Walter et al., 2018; Harkema et al., 2018b; Herrity et al., 2018; DiMarco et al., 2021) and transcutaneous (tSCS) SCS (Gad et al., 2018; Doherty et al., 2019; Hodgkiss et al., 2024; Engel-Haber et al., 2024).

To the best of our knowledge, this systematic review represents the most comprehensive synthesis of published evidence on spinal cord and spinal root stimulation for autonomic dysfunction after SCI. Although most contemporary SCS approaches target the dorsal roots or surface of the spinal cord, we also include systems that stimulate anterior roots, such as Brindley Sacral Anterior Root Stimulation (SARS) device. Including both paradigms allows for a more complete evaluation of neuromodulatory strategies across different spinal cord targets. In addition, we also examine stimulation parameters to synthesise stimulation strategies associated with greatest therapeutic benefit.

## 2 Methods

This review was registered on PROSPERO in April 2025.^1^ A literature search of OVID Medline, OVID Embase; and Web of Science was conducted on 20/09/24. Search terms were as follows: (Spinal Cord Stimulation OR Epidural Stimulation OR Transcutaneous Spinal Cord Stimulation OR TSCS OR Dorsal Root Stimulation OR Posterior Root Stimulation OR Lumbosacral Neuromodulation, Thoracic Neuromodulation, Cervical Neuromodulation, Sacral Anterior Root Stimulation OR Brindley) AND (Spinal Cord Injury OR SCI OR Paralysis OR Paraplegi* OR Tetraplegi* OR Quadriplegi*) AND (Autonomic Dysfunction OR Sympathetic OR Parasympathetic OR Autonomic Dysreflexia OR Neurogenic Bladder OR Overactive Bladder OR NLUTD OR Urinary Incontinence OR Urinary Retention OR Detrusor Overactiv* OR Detrusor Hyper-reflexia OR Dys-synergia OR Vesico-Ureteral Reflux OR Cystometr*y OR Urodynamic*s OR Neurogenic Bowel OR Bowel Motility OR Anorectal OR Faecal Incontinence OR Constipation OR Diarrhoea OR Manometry OR Sex* OR Sexual Dysfunction OR Erectile Dysfunction OR Anejaculation OR Vaginal Lubrication OR Sexual Arousal OR Orgasm OR Cardiovascular Dysfunction OR Orthostatic Hypotension OR Hypotension OR Hypertension OR Malignant HTN OR Blood Pressure OR Heart Rate OR Tachycardi* OR Bradycardi* OR Dysrhythmia OR Thermoregulation OR Temperature OR Hyperthermia OR Hypothermia OR Poikilothermia OR Fever OR Sweat* OR Hyperhidrosis OR Hypohidrosis OR Piloerection OR Respiratory Dysfunction OR Pulmonary Dysfunction OR Breath* OR Apnoea OR Inspirat* OR Expirat* OR Ventilat* OR Cough OR Lung Volume OR Diaphragm).

### 2.1 Eligibility criteria

Selection criteria were established by the PICOS (Population, Intervention, Comparison, Outcome, and Study design). Inclusion criteria: Adults with Spinal Cord Injury (≥18 years old); Spinal Cord Stimulation (eSCS, tSCS or SARS); outcome measures assessed before and after intervention, or if a comparison has been made between different groups (e.g., SCI-Controls vs. SCS or Sham-stimulation vs. SCS); studies with at least one quantitative outcome related to bladder, bowel, sexual, cardiovascular, respiratory or thermoregulatory function. Additional outcomes include stimulation parameters (level targeted, electrode configuration, frequency and intensity) and mapping strategies across studies. Exclusion criteria: Animal studies; Mixed Populations; Non-SCI, e.g., healthy controls or other neurological pathology; Computational Models, Alternative stimulation sites, e.g., peripheral nerve stimulation, sacral neuromodulation (SNM); Alternative types of electrical stimulation; Studies not reporting any quantitative outcomes; non-English articles; Conference Abstracts (non-peer reviewed material).

Three independent authors (P.U., C.H. & I.T.) performed screening of title, abstract and full text. The senior author (L.D.) resolved conflicts regarding the eligibility of studies. The screening and selection process was performed using the Rayyan® software.

### 2.2 Data extraction

Extracted data included study design, number of SCI participants, type of stimulation applied, device used, stimulation parameters (frequency, pulse-width, intensity, electrode configuration), duration of intervention, combination of interventions, and quantitative outcome measures representing autonomic dysfunction.

Quantitative data was synthesised into tables where 3 or more studies report the same outcome measure. Data was then synthesised in subsets, according to each autonomic dysfunction: (1) cardiovascular (resting blood pressure (BP) and heart rate (HR); BP post orthostatic challenge or AD challenge); (2) urinary (urodynamic measurements – bladder capacity, detrusor pressure, bladder compliance, voiding efficiency; continence; UTI rates; Neurogenic Bladder Symptom Score (NBSS)); (3) bowel (bowel management (BM) duration and frequency; episodes of incontinence and constipation; manometric data; Neurogenic Bowel Dysfunction Score (NBDS)); (4) sexual (erection and ejaculation rates; sexual function related questionnaires/scores).

For analysis, data specific to the different autonomic functions were then classified by (1) type of intervention (eSCS or tSCS or SARS); (2) intervention duration (acute single session vs. chronic intervention); (3) outcome measure (studies reporting comparable outcome measures, assessed using similar methods). Tables were created to summarise the range of stimulation parameters and electrode configurations utilised to date.

In cases where multiple publications reported identical outcomes from the same cohort, data was extracted from the most recent article. Additional data from the same cohort were included only if outcomes were assessed by a different method or if a distinct intervention was evaluated. Additionally, where results were not reported in the required format, we attempted to contact the relevant author listed. If this was not successful, we used a software ‘PlotDigitizer Version 3.1.6’ PlotDigitizer (2025) to estimate value from figures provided within the article.

### 2.3 Statistical analysis and risk of bias

Meta-analysis was not undertaken as part of this review due to small sample sizes, heterogeneity in outcome measures and reporting methods, and incomplete reporting of outcome measures. The included studies were also heterogeneous in design, comprising predominantly of case series and case reports, alongside cohort studies and a single randomised controlled trial. As no quantitative analysis or estimation of pooled effects was to be undertaken, no formal risk of bias assessment, to inform weighting, was performed. Nevertheless, the inherent limitations and biases of the included studies, were considered in the interpretation of findings.

## 3 Results

### 3.1 Article selection

**Figure 1:**
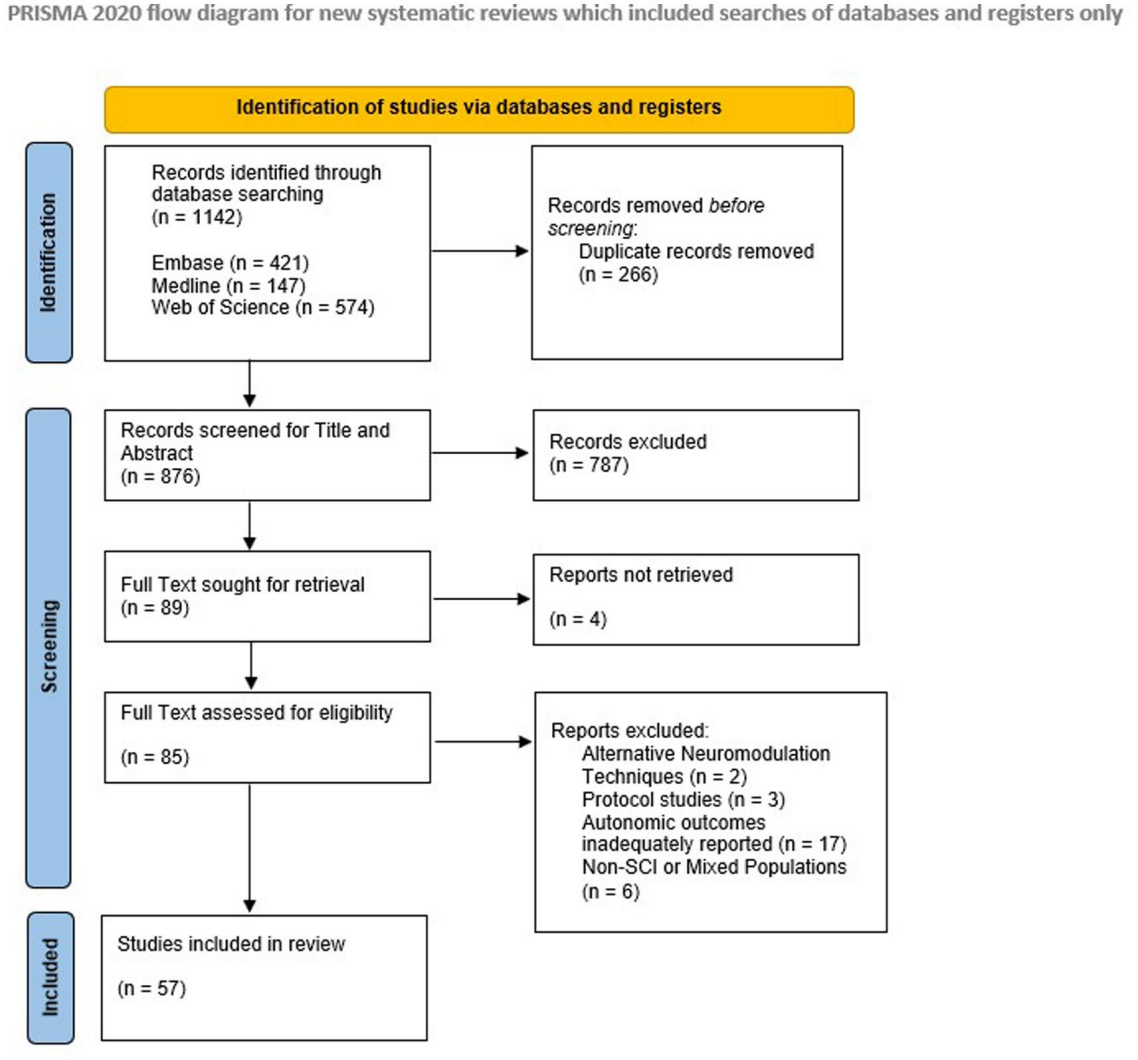
PRISMA flow chart (Page et al., 2021). PRISMA 2020 diagram has been adapted from the following source website: https://www.prisma-statement.org/prisma-2020-flow-diagram. Licence: https://creativecommons.org/licenses/by/4.0/.

Following the removal of duplicates (*n* = 266), the database search identified 876 articles. 787 were non-compliant with study criteria and were excluded following title and abstract screening. Eighty-five articles underwent full text screening: 4 articles could not be retrieved, and 28 were excluded. Fifty-seven articles were included in the review (Figure 1—PRISMA flow diagram). A summary of the articles included can be found in Table 1.

**Table 1:** Summary of all articles included in the systematic review, including number of participants with spinal cord injury (N SCI), clinical characteristics (level: cervical (C), Thoracic (T), Lumbar (L) or Sacral (S), ASIA Impairment Scale (AIS)), type of stimulation (stim) given (epidural (eSCS) or transcutaneous (tSCS) spinal cord stimulation or sacral anterior root stimulation with sacral deafferentation (SARS-SDAF) or without (SARS)), acute (single session, outcome measures comparing with and without stimulation) or chronic (long-term intervention (>1 session) with pre-post outcome measures) and autonomic functions investigated in outcome measures.

Article	*N*		Age (year)	SCI level	AIS				Stim type	Acute or chronic intervention	Concurrent therapy	Autonomic outcome measures
					Complete		Incomplete					
	M	F			A	B	C	D				
Engel-Haber et al. (2024)	8	0	30.2	C3-C7	4	3	1	0	tSCS	Acute	None	CVS
Solinsky et al. (2024)	1	1	25	T3, T5	2	0	0	0	tSCS	Acute	None	CVS
Hodgkiss et al. (2024)	4	0	44.3	C6-T4	3	1	0	0	eSCS (*n* = 2) tSCS (*n* = 2)	Acute	None	CVS
Kumru et al. (2023)	10	1	29.3	C3-C7	2	4	4	1	tSCS	Chronic – 30 min/day for 5 sessions	Inspiratory Muscle Training	Respiratory
Shackleton et al. (2023)	0	3	29.3	T4-T8	1	0	0	0	eSCS	Chronic – 24 h/day for 13 months	None	Bladder, Sexual
Samejima et al. (2023a)	2	1	34	C6-T5	1	2	0	0	eSCS	Acute	None	CVS
Rybka et al. (2024)	2	0	28	T4	1	0	0	0	eSCS	Chronic – 3–4 h/day for 2 months	None	Sexual
Gorgey et al. (2023)	1	0	27	C8	0	1	0	0	eSCS	Acute and Chronic – 1 h/day, 3/week for 15 weeks	Exoskeleton-assisted walking training	CVS
Kreydin et al. (2022)	2	2	32	C5-T6	1	0	0	0	tSCS	Acute and Chronic: 1 h/day, 5 days/week for 5 weeks	None	Bowel
Kandhari et al. (2022a)	9	1	32.9	T2-T12	1	0	0	0	eSCS	Chronic: 12–16 h/day for 2 months	Activity Based Therapy (2 h/day)	Bowel, Sexual
Kandhari et al. (2022b)	1	1	25.5	C3–4	1	0	0	0	eSCS	Acute	None	Respiratory
Samejima et al. (2022)	2	0	32.1	C4–6	0	0	0	1	tSCS	Chronic:, 2 h/day for 2 months	Locomotor Training	Bladder, Bowel
Herrity et al. (2022)	6	1	32.1	C3-T2	2	5	0	0	eSCS	Acute	None	Bladder, CVS
Sachdeva et al. (2021)	1	0	37	C4	1	0	0	0	tSCS	Acute	None	CVS
Squair et al. (2021)	1	0	38	C5	1	0	0	0	eSCS	Acute	None	CVS
Herrity et al. (2021)	8	2	28.6	C2-T4	6	4	0	0	eSCS	Acute and Chronic: 160 1-h sessions	Locomotor Training	Bladder, CVS
DiMarco et al. (2021)	5	0	37	C3-T1	1	0	0	0	eSCS	Chronic – 5–10 min, 2–3/day for 21 weeks	None	Bowel, Respiratory
Legg Ditterline et al. (2021)	3	1	30.8	C4	3	1	0	0	eSCS	Chronic – 89 2-h sessions	None	CVS
Beck et al. (2021)	2	0	31.5	T3-T6	1	0	0	0	eSCS	Chronic – 12 months	Multi-modal rehabilitation	Bladder
Gad et al. (2020)	1	0	39	C5	1	0	0	0	tSCS	Chronic – 1-h/day, 5 days/week for 2 weeks	None	Respiratory
DiMarco et al. (2020)	10	0	40	C2-T1	NR				eSCS	Chronic: 5–10 min, 2–3/day for 20 weeks	None	Respiratory
Legg Ditterline et al. (2020)	3	1	40	C4	3	1	0	0	eSCS	Chronic – 89 2-h sessions	None	CVS
Nightingale et al. (2019)	1	0	33	C5	0	1	0	0	eSCS	Acute	None	CVS
DiMarco et al. (2019)	3	0	33	C2-C4	2	1	0	0	eSCS	Chronic: 5–10 min, 2–3/day for 28 weeks		Respiratory
Doherty et al. (2019)	7	0	53.1	C5-L1	3	0	1	3	tSCS	Acute	None	Bladder
Darrow et al. (2019)	0	2	50	T4-T8	1	0	0	0	eSCS	Chronic – daily for 5 months	None	CVS, Bladder, Bowel, Sexual
Phillips et al. (2018)	4	1	28	C5-T2	3	1	0	0	tSCS	Acute	None	CVS
Herrity et al. (2018)	1	0	31	C5	0	1	0	0	eSCS	Acute	Locomotor Training	Bladder
Walter et al. (2018)	1	0	32	C5	0	1	0	0	eSCS	Acute and Chronic – 1 month	for bowel movement	Bowel
Gad et al. (2018)	4	3	39	C3-T11	4	1	2	0	tSCS	Acute	None	Bladder
Zaer et al. (2018)	154	133	49	C (*n* = 136); T (*n* = 148); L (*n* = 3)	242	39	6	0	SARS-SDAF	Chronic – median follow 13 years (range 1–25 years)	NR	Bladder, Sexual
DiMarco et al. (2018)	1	0	50	C4	NR				eSCS	Chronic: 5–10 min, 2–3/day for 8 weeks	None	Respiratory
Harkema et al. (2018a)	3	1	30.8	C4	3	1	0	0	eSCS	Chronic – 89 2-h sessions	None	CVS
Aslan et al. (2018)	7	0	26.7	C5-T4	4	3	0	0	eSCS	Acute	None	CVS
Harkema et al. (2018a)	3	1	30.8	C4	3	1	0	0	eSCS	Acute	None	CVS
West et al. (2018)	1	0	30	C5	0	1	0	0	eSCS	Acute	None	CVS
Krebs et al. (2017)	59	52	50	Tetra (*n* = 39); Para (*n* = 72)	NR				SARS-SDAF	Chronic – median 11.7-year follow up (range 5–24.9 years)	NR	Bladder
Castaño-Botero et al. (2016)	95	9	38	C (*n* = 34); T (*n* = 68); L (*n* = 2);	96	8	0	0	SARS-SDAF	Chronic – NR; post-operative	NR	Bladder, Bowel Sexual, CVS
Rasmussen et al. (2015)	145	132	49	C (*n* = 131); T (*n* = 143); L (*n* = 3);	234	38	5	0	SARS-SDAF	Chronic – median follow 13 years (range 1–25 years)	NR	Bowel
Krasmik et al. (2014)	81	56	40	C (*n* = 53); T (*n* = 81); L (*n* = 3)	132	4	1	0	SARS-SDAF	Chronic – mean follow up on 14.8 + −5.3 years	NR	Bladder, CVS
DiMarco et al. (2014)	8	2	35.6	C3-T5	NR				eSCS	Chronic: mean 4.6 years	None	Respiratory
DiMarco et al. (2009)	8	1	41	C3-C6	NR				eSCS	Chronic: 5–10 min, 2–3/day for 12 weeks		Respiratory
Vallès et al. (2009)	9	9	39	C (*n* = 4); T (*n* = 14); L-S (*n* = 1)	14	1	3	0	SARS-SDAF	Chronic – evaluate 12 months after	None	Bowel
DiMarco et al. (2006)	1	0	52	C5-C6	NR				eSCS	NR	None	Respiratory
DiMarco et al. (2005)	3	1		C2-C4	NR				eSCS	Chronic: 20 weeks	Phrenic Nerve Pacing	Respiratory
Kirkham et al. (2002)	5	0	37.2	T3-T10	5		0		SARS	Acute	None	Bladder, Sexual
van der Aa et al. (1999)	33	5	35	C4-T12	5		5		SARS-SDAF	Chronic – 3 months to 12 years of follow-up	None	Bladder, Bowel
Egon et al. (1998)	68	28	32.8	C (*n* = 41), T (*n* = 43), Below T10 (n – 12)	79		17		SARS-SDAF	Chronic – 2 days Post-operatively, follow-up mean 5.4–5.8 years	None	Bladder, Bowel, Sexual
Schurch et al. (1997)	3	7	28.7	C5-T6	10		0		SARS-SDAF	Chronic – 4 days Post-operatively, follow-up mean 5.4–5.8 years	None	Bladder
Van Kerrebroeck et al. (1997)	29	23	32.9	C (*n* = 11), T (*n* = 41)	52		0		SARS-SDAF	Chronic – 6 months follow-up	None	Bladder, Bowel, Sexual
Van Kerrebroeck et al. (1996)	29	23	32.9	C (*n* = 11), T (*n* = 41)	52		0		SARS-SDAF	Chronic – 6 months follow-up	None	Bladder, Bowel, Sexual
van der Aa et al. (1995)	14	3	37.2	C5-T10	17		0		SARS-SDAF	Chronic – follow up 1–6 years	None	Bladder, Sexual
Koldewijn et al. (1994)	14	13	34	C4-T12	27		0		SARS-SDAF	Chronic – results at 6 months	None	Bladder, Sexual
Sahni et al. (1991)	31	2	40	C4-T10	NR				eSCS	Acute (post implantation)	None	Bladder
MacDonagh et al. (1990)	9	3	33	C (*n* = 2); T (*n* = 10)	12		0		SARS-SDAF	Chronic – mean follow-up 26.3 months	None	Bowel
Arnold et al. (1986)	5	1		T3-T11	NR				SARS	Acute (post-implantation) and Chronic – 18-month follow-up	None	Bladder, Sexual
Richardson et al. (1979)	5	0	21.2	T2-L2	NR				eSCS	Chronic – 2-yer follow-up	None	CVS

NR, not reported.

Most studies investigated epidural (*n* = 30) and/or transcutaneous (*n* = 10) SCS, predominantly in the form of case series (*n* = 18), case reports (*n* = 10), alongside several small prospective cohort studies (*n* = 11) and a single randomised controlled trial (*n* = 1). eSCS trials reported on a total of 151 participants (130 males; 21 females) with an average age of 34 years. tSCS trials reported on 48 participants, (40 males; 8 females) with an average age of 34 years. Fifteen studies examined sacral anterior root stimulation with anterior root deafferentation (SARS-SDAF), made up of prospective cohort studies or case series’ (*n* = 10), and some, more recent, retrospective analyses of large cohorts (*n* = 5), reporting on a total of 1,238 participants (742 males; 496 females), with an average age of 37 years. Only two trials – both case series’—studied SARS (without deafferentation), reporting on 11 participants (11 males; 1 female), with an average age of 37 years. One of these trials not performing deafferentation, stimulated both the Sacral Posterior and Anterior Roots, denoted as ‘SPARS’ (Kirkham et al., 2002). Across all trials, where reported, SCI levels ranged from C2 to L2. Amongst eSCS trials, all participants had either AIS A (59%) or B (41%) injuries. For tSCS trials, participants mostly had AIS A injuries (49%); the remaining having AIS B (21%), C (19%) or D (11%) injuries. Amongst SARS-SDAF trials, the vast majority had AIS A injuries (87%) with the remaining having AIS B (11%) or C (2%) injuries.

Across both eSCS and tSCS, electrode arrays or surface electrodes were almost invariably implanted or applied at the T10-T11/12 vertebral levels to access the L1-S1 spinal segments (Table 1). A summary of reported stimulation parameters and electrode configurations (eSCS) is provided in Supplementary Tables S1, S2. For eSCS, stimulation is biphasic in nature. Pulse widths commonly used were ~500 μs, with 450 μs frequently reported in the literature by the Herrity and Harkema group (Harkema et al., 2018a; Harkema et al., 2018b; Legg Ditterline et al., 2021; Herrity et al., 2021; Herrity et al., 2022). Other studies reported pulse widths between 200 and 500 μs (Supplementary Table S1). Stimulation Frequency was variable, though 30–60 Hz has been applied most frequently for both motor and autonomic modulation. tSCS studies employed longer pulse widths, most often 1 ms, and in one case, 2 ms (Sachdeva et al., 2021). Frequencies range from 0.5–120 Hz, with 30 Hz being particularly common, and more recently applied in 5 or 10 kHz bursts. tSCS has been commonly applied as monophasic waves, although three studies applied biphasic tSCS (Sachdeva et al., 2021; Kreydin et al., 2022; Solinsky et al., 2024). SARS leads are typically implanted over the S2-S4/5 anterior roots, with stimulation parameters not commonly reported.

Of the 57 articles included in the review, 22 investigated autonomic outcomes related to cardiovascular function (eSCS = 15; tSCS = 5; SARS-SDAF = 2), 23 investigated outcomes related to urinary function (eSCS = 7; tSCS = 3; SARS-SDAF = 11; SARS = 2), 14 investigated outcomes related to bowel function (eSCS = 4; tSCS = 2; SARS-SDAF = 8); 12 investigated outcomes related to sexual function (eSCS = 4; SARS-SDAF = 7; SARS = 2).

### 3.2 Cardiovascular function

Outcomes related to cardiovascular (CVS) function were reported in 22 articles using eSCS (*n* = 15), tSCS (*n* = 5) and/or SARS (*n* = 2), involving a total of *n* = 54 (eSCS), *n* = 20 (tSCS) or *n* = 232 (SARS) participants (Table 1). Injury level ranged from C2-L2 with most injuries at the cervical level or high thoracic level. All injured were AIS A/B, except for 5 participants with AIS C in a retrospective study of SARS (Krasmik et al., 2014). Most studies reported the acute effects of stimulation, with only four studies exploring longer term effects (Legg Ditterline et al., 2021; Krasmik et al., 2014; Castaño-Botero et al., 2016; Gorgey et al., 2023).

Using eSCS, the lumbosacral nerve roots (L1-S1) were primarily targeted (Aslan et al., 2018; West et al., 2018; Darrow et al., 2019; Harkema et al., 2018b; Herrity et al., 2021; Herrity et al., 2022; Legg Ditterline et al., 2021; Samejima et al., 2023a; Gorgey et al., 2023; Hodgkiss et al., 2024), using electrode configurations that typically targeted the upper segments of the lumbar cord (Harkema et al., 2018b; Darrow et al., 2019; Legg Ditterline et al., 2021). One study targeted the slightly higher thoracolumbar roots (T10-L2) (Squair et al., 2021). Similarly, tSCS primarily targeted the lumbosacral nerve roots (T10-L2 vertebral level) (Engel-Haber et al., 2024; Hodgkiss et al., 2024; Solinsky et al., 2024), with one study placing electrodes at the T7-T8 vertebral level (Phillips et al., 2018). Two studies also evaluated cervical tSCS (Samejima et al., 2022; Engel-Haber et al., 2024), part of a multi-site stimulation protocol in one of the studies (Samejima et al., 2022). Stimulation was commonly applied at frequencies between 30 and 60 Hz (West et al., 2018; Aslan et al., 2018; Harkema et al., 2018b; Darrow et al., 2019; Nightingale et al., 2019; Legg Ditterline et al., 2021; Samejima et al., 2023a), although others reported frequencies included 2, 25, 85, 120 and 300 Hz (Aslan et al., 2018; Squair et al., 2021; Herrity et al., 2022; Gorgey et al., 2023; Solinsky et al., 2024) (Supplementary Table S1). With tSCS, some groups also used short (1 ms) high frequency (5 or 10 kHz) bursts of stimulation applied at 30 Hz (Engel-Haber et al., 2024; Hodgkiss et al., 2024). tSCS was applied as monophasic waves, except in 2 studies applying biphasic stimulation evaluating neuromodulation of AD (Sachdeva et al., 2021; Solinsky et al., 2024).

Outcome measures that were adequately reported on and evaluated were: Systolic Blood Pressure (SBP), Diastolic Blood Pressure (DBP), and Heart Rate (HR) at rest or in response to a haemodynamic challenge, i.e., aiming to trigger orthostatic drops or autonomic dysreflexia. Although Low Frequency (LF) and High Frequency (HF) components of the HR and BP Variability spectra were not originally specified as outcomes, we observed four studies reporting relevant data. Consequently, these outcomes measures were included in the analysis, as they met the threshold of three or more studies reporting on an outcome measure, outlined in our methods.

**Figure 2:**
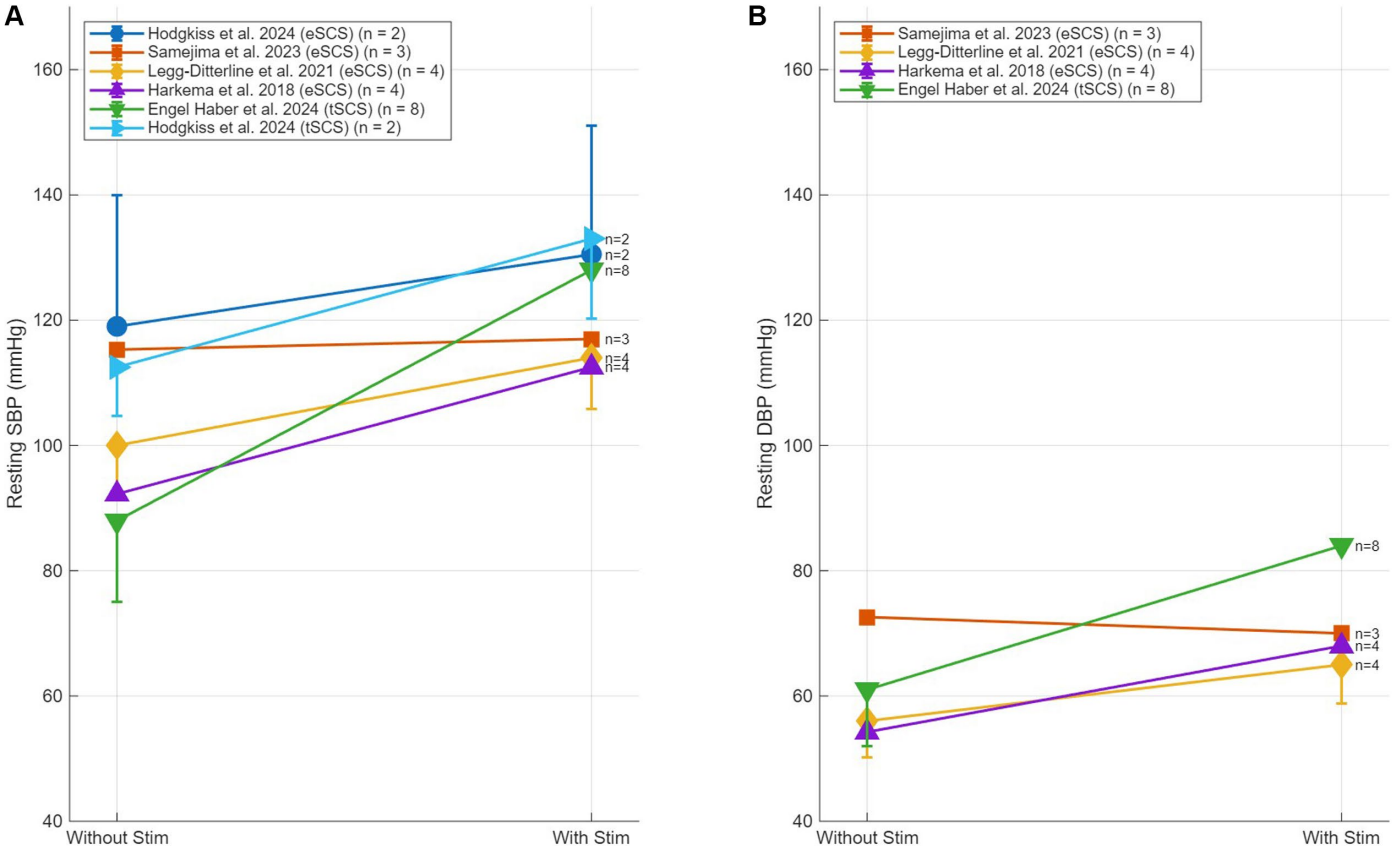
Mean (SD) resting systolic blood pressure (SBP) (panel **A**) and diastolic blood pressure (DBP) (panel **B**) without stimulation (stim) and with acute stimulation across studies using epidural (eSCS) or transcutaneous (tSCS) spinal cord stimulation. Sample sizes and modality of stimulation have been noted in the legend.

Five eSCS studies (*n* = 14) and two tSCS studies (*n* = 10) investigated the acute effect of SCS on resting BP and HR (Table 2). All trials showed an acute rise in resting SBP, and three of four reporting acute rise in DBP, with SCS targeting the lumbosacral roots (Figure 2). One eSCS study reported no significant differences in SBP when eSCS targeted the upper (rostral) compared with caudal lumbosacral roots (Aslan et al., 2018). One case series systematically mapped the effects of tSCS on resting BP between cervical (C3/4—C7/T1), upper thoracic (T1/2), and lumbosacral (T11/12, L1/2, S1/2) vertebral levels (Engel-Haber et al., 2024). Lumbosacral stimulation consistently elevated resting BP, whereas cervical and upper thoracic stimulation did not. Post-hoc analyses indicated that only stimulation at T11/12 vertebral level yielded significant increases in SBP (+40 ± 19 mmHg) and DBP (+23 ± 11 mmHg) compared with cervical stimulation (*p* < 0.05) (Engel-Haber et al., 2024).

**Table 2:** Reported resting blood pressure (BP) and heart rate (HR) at baseline (without stimulation), during acute application of stimulation (stim) and post-intervention for chronic trials that applied stimulation using epidural (eSCS) or transcutaneous (tSCS) spinal cord stimulation.

Article	Stim type	Level targeted	Stim frequency (Hz)	*N*	Resting BP (mmHg)			Resting HR (bpm)		
					Baseline	Acute (with stim)	Chronic	Baseline	Acute (with stim)	Chronic
Hodgkiss et al. (2024)	eSCS (map)	T10-12 or T11-L1 (Vertebral)	30 or 300	2	119 ± 21 (SBP)	130.5 ± 20.5 (SBP)	NR	NR		
Samejima et al. (2023a) and Samejima et al. (2023b)	eSCS	T10-12 (Vertebral)	17–35	3	115.3 (SBP)	117.0 (SBP)	NR	62.3	62.3	NR
					72.6 (DBP)	70.0 (DBP)				
Legg Ditterline et al. (2020)	eSCS (map)	L1-S1 (spinal)	30–60	4	100 ± 7.5 (SBP)	114.0 ± 8.1 (SBP) (*p* < 0.05)	106 ± 7.6 (SBP) (*p* > 0.05)	56.0 ± 4	53 ± 4.5 (*p* = 0.502)	54 ± 4 (*p* > 0.05)
					56.0 ± 5.8 (DBP)	65.0 ± 6.2 (DBP) (*p* < 0.05)				
Nightingale et al. (2019)	eSCS (map)	T11–L1 (Vertebral)	35	1	NR	NR (change of 14 mmHg)	NR	NR		
Harkema et al. (2018a)	eSCS (map)	T11–L1 (Vertebral)	30–60	4	92.3 (SBP)	112.5 (SBP) (*p* < 0.05)	NR	NR		
					54.3 (DBP)	68.0 (DBP) (*p* < 0.05)				
Engel-Haber et al. (2024)	tSCS	C3/4 (Vertebral)	30 (5 kHz burst)	8	93 ± 12 (SBP)	100 (SBP)	NR	73 ± 10	73	NR
					69 ± 9 (DBP)	73 (DBP)				
		C6/7 (Vertebral)	30 (5 kHz burst)	8	93 ± 12 (SBP)	107 (SBP)		73 ± 7	73	NR
					65 ± 7 (DBP)	74 (DBP)				
		T1/2 (Vertebral)	30 (5 kHz burst)	8	91 ± 14 (SBP)	102 (SBP)		83 ± 13	80	NR
					66 ± 10 (DBP)	72 (DBP)				
		T11/12 (Vertebral)	30 (5 kHz burst)	8	88 ± 13 (SBP)	128 (SBP)		75 ± 10	59	NR
					61 ± 9 (DBP)	84 (DBP)				
		L1/L2 (Vertebral)	30 (5 kHz burst)	8	92 ± 11 (SBP)	119 (SBP)		75 ± 8	68	NR
					67.0 ± 7 (DBP)	81.0 (DBP)				
Hodgkiss et al. (2024)	tSCS	T11-L1 (vertebral)	30 (5 kHz burst)	2	112.5 ± 7.8 (SBP)	133.0 ± 12.7 (SBP)	NR	76.8	67	NR

Spinal or vertebral level targeted with stimulation and stimulation parameters are also provided, with studies that performed mapping specifically for cardiovascular function indicated (map). DBP, diastolic blood pressure; N, number; NR, not reported; P, participant; SBP, systolic blood pressure.

**Figure 3:**
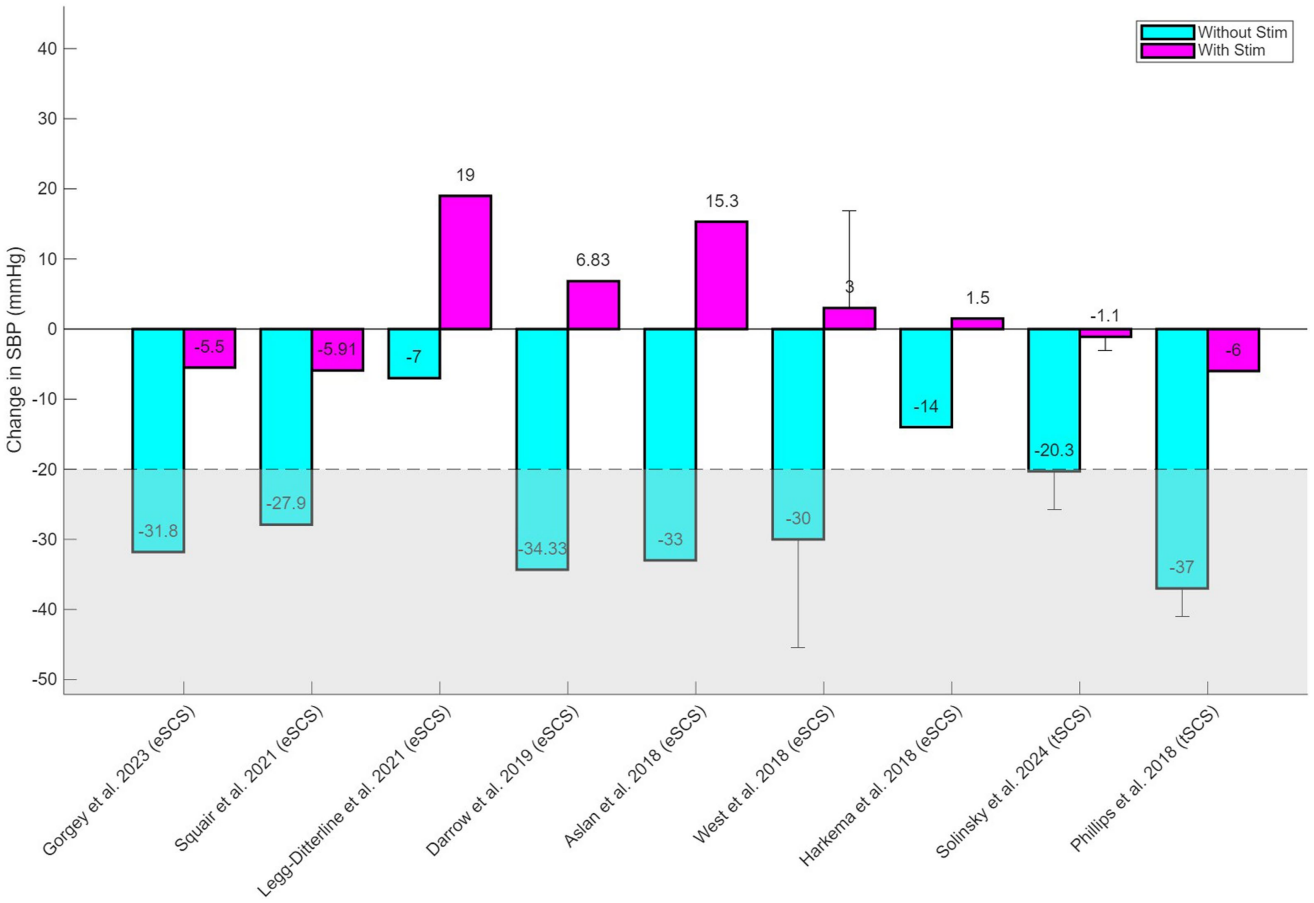
Mean (SD) change in systolic blood pressure (SBP) during orthostatic challenge without stimulation (light blue) and with acute stimulation (pink) across studies using epidural (eSCS) or transcutaneous (tSCS) spinal cord stimulation. The grey area below the dashed line indicates the threshold for orthostatic hypotension.

Six eSCS studies (*n* = 11) and two tSCS studies (*n* = 7) investigated the acute effect of SCS during orthostatic stress (Table 3). All studies reported amelioration of drop in SBP post orthostatic challenge with acute eSCS and tSCS application (Table 3; Figure 3). Seven of these 8 SCS studies (*n* = 15) reported improvements in SBP drops, such that patients no longer met the criteria for OH, clinically defined as a drop in SBP of >20 mmHg (Freeman et al., 2011), and three studies (*n* = 8) reported that SCS elevated SBP above baseline level (pre-orthostatic stress test) (Aslan et al., 2018; Darrow et al., 2019; Legg Ditterline et al., 2021). One study reported that only patients with baseline OH demonstrated notable increases in BP with eSCS (Aslan et al., 2018). Two studies investigated the chronic application of eSCS on OH (Legg Ditterline et al., 2021; Gorgey et al., 2023). Of these, only one study (*n* = 4) reported that effects on OH were maintained in the longer term and in the absence of stimulation (Legg Ditterline et al., 2021). One eSCS case report, where the electrode array was implanted at the T10-T11 vertebral levels reported that stimulation at the rostral end of the array, targeting the caudal thoracic roots (T10-T12) produced a controlled increase in SBP of 39.9 ± 3.4 mmHg, whereas stimulation at the middle and caudal portions of the array (over lumbosacral roots), resulted in more modest elevations of 13.8 ± 8.4 mmHg and 4.5 ± 3.8 mmHg, respectively (Squair et al., 2021) (Supplementary Table S2). Similarly, two studies reported that targeting mid-lower lumbosacral roots impaired the ability to correct BP during resting or OH, compared to targeting the upper lumbar roots (Darrow et al., 2019; Hodgkiss et al., 2024). With regards to stimulation frequency, one study directly compared the acute effects of tSCS at 30 Hz, 120 Hz, and 30 Hz applied in 5 kHz bursts, reporting that 120 Hz was most effective in correcting BP drops during OH, whilst 30 Hz with 5 kHz was less effective and 30 Hz alone was ineffective (Solinsky et al., 2024).

**Table 3:** Change in systolic blood pressure (SBP) during orthostatic challenge (mmHg) at baseline (without stimulation), during acute application of stimulation (stim) and post-intervention for chronic trials that applied stimulation using epidural (eSCS) or transcutaneous (tSCS) spinal cord stimulation.

Article	Stimulation type	Test	Level targeted	Stim frequency (Hz)	*N*	Change in SBP during orthostatic challenge (mmHg)		
						Baseline	Acute (with stim)	Chronic
Gorgey et al. (2023)	eSCS	Tilt-Table Assessment	T10-T12 (Vertebral)	25	1	−31.8	−5.5	−32.5
Squair et al. (2021)	eSCS (map)	Tilt-Table Assessment	T10-11 (Vertebral) targeting T10-L1 (Spinal) Config A^^^	120	1	−27.9	−5.9	NR
Legg Ditterline et al. (2020)	eSCS (map)	Sit-up Test	T11-L1 (Vertebral) – L1-S1 (Spinal)	30–60	4	−7	19 (*p* < 0.0001)	8 (*p* = 0.0004)
Darrow et al. (2019)	eSCS (map)	Tilt-Table Assessment	T12 (Vertebral)	50	1	−34.3*	6.8* (*p* < 0.001)	NR
Aslan et al. (2018)	eSCS	Sit-up Test and Standing	T11-L1 (Vertebral) – L1-S1 (Spinal)	15–30	3	−33 ± 15.4	15.3 ± 13.9	NR
West et al. (2018)	eSCS (map)	Tilt-Table Assessment	T11-L1 (Vertebral) – L1-S1 (Spinal)	35	1	−30*	3*	NR
Solinsky et al. (2024)	tSCS	Valsalva Manoeuvre	T10-T11 (Vertebral)	120	2	−20.3 ± 5.5	−1.1 ± 2.0	NR
Phillips et al. (2018)	tSCS	Sit-up Test	T7-T8	30	5	−37 ± 4*	−6 (*p* < 0.05)	NR

Spinal or vertebral level targeted with stimulation and stimulation parameters are also provided, with studies that performed mapping specifically for cardiovascular function indicated (map). *Estimated using PlotDigitizer. ^For configs, see Supplementary Table S2. N, number; NR, not reported; P, participant.

Three eSCS studies (*n* = 20) and two tSCS studies (*n* = 3) explored the acute effect of SCS during AD challenges (Table 4). Three of these studies (*n* = 8) showed that SCS mitigated AD in response to an artificial noxious stimulus, whilst maintaining a stable HR: two studies applied eSCS, targeting the mid-caudal lumbosacral roots (L3/4 and below) (Herrity et al., 2022; Samejima et al., 2023a) and the third study applied tSCS at the T7/T8 vertebrae (Sachdeva et al., 2021). Of the remaining two studies, one applied eSCS to the lumbosacral roots and found no effect. The other applied tSCS at T10-11 and reported exacerbation of AD with consistent elevations in BP and reduction in HR (Solinsky et al., 2024). They further compared acute tSCS at 30 Hz, 120 Hz, and 30 Hz applied in 5 kHz bursts, reporting that 120 Hz most significantly exacerbated AD, followed by 30 Hz with 5 kHz and 30 Hz alone (Solinsky et al., 2024). As noted, the two tSCS studies report conflicting effects: one reported attenuation of SBP elevation (Sachdeva et al., 2021), whilst the other reported a significant exacerbation (Solinsky et al., 2024). Notably, the stimulation was applied at different vertebral levels, T7-T8 in the former and T10-T11 in the latter. Three chronic studies reported on the occurrence of AD episodes. One reported that 2–3 h of daily eSCS over 2 years led to complete resolution of AD episodes in 4/5 patients (Richardson et al., 1979). A further two retrospective studies both reported that after implantation of a SARS device, there was a significant reduction in incidence of AD from 61.3 to 2.2% (*n* = 84, *p* < 0.001) and 66 to 6% (*n* = 104, *p* < 0.001) across their participants, respectively (Krasmik et al., 2014; Castaño-Botero et al., 2016).

**Table 4:** Change in blood pressure (BP) and heart rate (HR) during autonomic dysreflexia (AD) challenge at baseline (without stimulation), during acute application of stimulation (stim) and post-intervention for chronic trials that applied stimulation using epidural (eSCS) or transcutaneous (tSCS) spinal cord stimulation.

Article	Stim type	Test	Level targeted	Stim frequency (Hz)	*N*	Change in BP (mmHg)			Change in HR (bpm)		
						Baseline	Acute (with stim)	Chronic	Baseline	Acute (with stim)	Chronic
Samejima et al. (2023a) and Samejima et al. (2023b)	eSCS	DARS	T10 − 11 or T11-12 (Vertebral)	17–35	3	26.3 (SBP)	12.3 (SBP)	NR	−11	−4.6	NR
Herrity et al. (2022)	eSCS	Bladder Distention	T11-L1 (Vertebral) – L1-S1 (Spinal)	60–85	4 (ISC)	NR (Mean SBP = 157 ± 7)	NR (Mean SBP = 121 ± 13) (−36 ± 9.5 (*p* = 0.043))	NR	NR	NR	NR
					3 (SPC)	NR (Mean SBP = 156 ± 4)	NR (Mean SBP = 139 ± 15) (−29.8 ± 24.0 (*p* = 0.1651))	NR	NR	NR	NR
Herrity et al. (2021)	eSCS	Bladder Distention	T11-L1 (Vertebral) – L1-S1 (Spinal)	NR	10	22 ± 20 (SBP)	NR	25 ± 11 (SBP)	NR	NR	NR
Solinsky et al. (2024)	tSCS	Foot Cold Pressor	T10–T11	30, 120 and 30 (5 kHz burst)	2	5 (MAP)	27.7 ± 4.5 (MAP)	NR	−2	−6.3 ± 1.3	NR
				120	2	P1: 3 P2: 7 (MAP)	P1: 21 P2: 19 (MAP)	NR	P1: −1 P2: −3	P1: −6 P2: −11	NR
				30	2		P1: 9 P2: 18 (MAP)	NR		P1: −6 P2: −5	
				30 (5 kHz burst)	2		P1: 9 P2: NR (MAP)	NR		P1: −2 P2: −9	
	tSCS	Bolus Phenylephrine	T10–T11	30, 120 and 30 (5 kHz burst)	2	6.8 ± 1.1 (MAP)	27.0 ± 2.4 (MAP) (*p* < 0.001)	NR	NR	NR	NR
				120	1	4 (MAP)	44 (MAP)				
				30	1		22 (MAP)				
				30 (5 kHz burst)	1		34 (MAP)				
Sachdeva et al. (2021)	tSCS	DARS	T7–T8	30	1	29 ± 5 (SBP)	5 ± 3 (DBP)	NR	−4 ± 1	−1 ± 0.5	NR

Spinal or vertebral level targeted with stimulation and stimulation parameters are also provided, with studies that performed mapping specifically for cardiovascular function indicated (map). DBP, diastolic blood pressure; IC, intermittemt catheter; MAP, mean arterial pressure; N, number; NR, not reported; P, participant; SBP, systolic blood pressure; SC, suprapubic catheter.

Three eSCS studies (*n* = 8) and one tSCS study (*n* = 2) investigated the effects of SCS on HR Variability (HRV) and BP Variability (BPV) spectra, either during orthostatic stress (Legg Ditterline et al., 2021; Gorgey et al., 2023) or AD (Solinsky et al., 2024; Samejima et al., 2023a) (Table 5). During orthostatic stress, SCS was associated with significant increases in both LF and HF HRV power, with a predominance of the LF power band (Legg Ditterline et al., 2021, Gorgey et al., 2023). These changes were accompanied by increased baroreflex effectiveness and stronger negative cross-spectrum correlations between LF systolic BP and HR (Legg Ditterline et al., 2021). In contrast, LF BPV changes were either not reported or showed minimal effects with acute or chronic stimulation interventions (Legg Ditterline et al., 2021; Gorgey et al., 2023). During AD challenges, findings were varied. Samejima et al. (2023a) and Samejima et al. (2023b) reported a reduction in LF BPV power with SCS, whereas Solinsky et al. (2024) reported increases in LF BPV and HF HRV, although not achieving statistical significance. Cross-spectrum relationships between BP and HR were either unchanged (Solinsky et al., 2024) or not reported (Samejima et al., 2023a).

**Table 5:** Effect of stimulation on heart rate variability (HRV) and blood pressure variability (BPV) in trials that applied stimulation using epidural (eSCS) or transcutaneous (tSCS) spinal cord stimulation.

Article	Stim type	*N*	Length of intervention	Level targeted	Stim freq (Hz)	Outcomes reported during	Effect of stimulation on HRV and BPV
Gorgey et al. (2023)	eSCS	1	Acute and Chronic (after 15 weeks exoskeleton training with eSCS 3/weeks)	T10-T12 (Vertebral)	25	Orthostatic Challenge – Tilt Table Test	Orthostatic stress reduced absolute LF and HF power in HRV. Application of eSCS increased power of both LF and HF bands, with LF band dominance with stimulation at both assessment points. By the second assessment, parasympathetic (HF) activity predominated in the supine position regardless of stimulation, but LF power again became dominant upon tilt. Cross-Spectrum heart rate and blood pressure relationship was not reported.
Legg Ditterline et al. (2021)	eSCS (map)	4	Acute and Chronic (after 89 days of 2 h of daily eSCS)	L1-S1 (spinal)	30–60	Orthostatic Challenge – Sit-up Test	Significant increases to both LF and HF HRV and baroreflex effectiveness both acutely and was sustained after a chronic intervention. Significantly stronger negative cross-correlation between LF SBP and HR acutely with SCS but not sustained after the chronic SCS intervention. There were no significant increases in LF BPV power band either acutely or after chronic intervention.
Solinsky et al. (2024)	tSCS	2	Acute	T10-T11	120	AD Challenge – Foot Cold Pressor or Bolus Phenylephrine	Stimulation appeared to increase LF BPV and HF HRV powers in those with SCI, this did not achieve significance. There was no reported significant change in HR and BP cross-spectrum relationships with stimulation.
Samejima et al. (2023a) and Samejima et al. (2023b)	eSCS	3	Acute	T10-11 or T11-12 (Vertebral)	17–35	AD Challenge – Digital Anorectal Stimulation	In the absence of eSCS, AD challenge resulted in a high LF band power in BPV, concomitant with the elevation of SBP during DARS. However, acute SCS resulted in decreased LF band power during AD challenge. HRV and their cross-spectrum relationships were not reported.

Spinal or vertebral level targeted with stimulation and stimulation parameters are also provided, with studies that performed mapping specifically for cardiovascular function indicated (map). AD, autonomic dysreflexia; HF, high frequency; LF, low frequency; N, number.

### 3.3 Urinary

Outcomes related to bladder function were reported in 23 articles applying eSCS (*n* = 7), tSCS (*n* = 3) and/or SARS (*n* = 13), involving a total of *n* = 58 (eSCS), *n* = 16 (tSCS) or *n* = 942 (SARS) participants (Table 1). eSCS participants (*n* = 58) either had SCI AIS A/B, ranging from level C2-T10. The majority were male (*n* = 48) with ages ranging from 28.6–50.0 years old. tSCS participants (*n* = 16), were also predominantly male (*n* = 13), with ages ranging from 38.0–50.0 years old. Injuries were AIS A-D ranging from level C3-L1. SARS participants (*n* = 942) were mostly male (*n* = 589), consisting of mostly cervical and thoracic injuries, and majority AIS A (*n* = 717), with ages ranging between 28.7–50.0 years old (Table 1).

Of the studies that applied eSCS or tSCS, the lumbosacral region was always targeted except for a study applying multi-site tSCS across the C3-L1 vertebral levels (Samejima et al., 2022). Overall, eSCS trial parameters ranged from 5–400 Hz, to 200–500 μS (Sahni et al., 1991; Herrity et al., 2018; Darrow et al., 2019; Herrity et al., 2022). To date, only one group has systematically mapped for eSCS electrode configurations to specifically target bladder-related networks, utilising pulse width of 450 μS and frequences between 20–85 Hz (Herrity et al., 2022), and 5–60 Hz (Herrity et al., 2018). tSCS studies applied either 15 Hz or 30 Hz (Gad et al., 2018; Doherty et al., 2019; Samejima et al., 2022), with one group applying stimulation along with a 10 kHz carrier frequency (Samejima et al., 2022). tSCS was applied at pulse widths between 200 and 1,000 μS. SARS trials stimulate the S2-S4 anterior toots, with one study also stimulating the sacral posterior (SPARS) (Kirkham et al., 2002). Eleven out of 13 SARS studies additionally performed deafferentation of the posterior sacral (S2–S4) roots (SARS-SDAF). In cases where deafferentation was performed, outcomes were not classified as acute, since any immediate changes represent alterations in bladder dynamics due to deafferentation rather than acute stimulation.

Outcome measures reported include bladder capacity, detrusor storage pressure, bladder compliance, voiding efficiency (%), post-void residual, Neurogenic Bladder Symptom Score, alongside urinary incontinence and UTI rate or occurrence.

**Figure 4:**
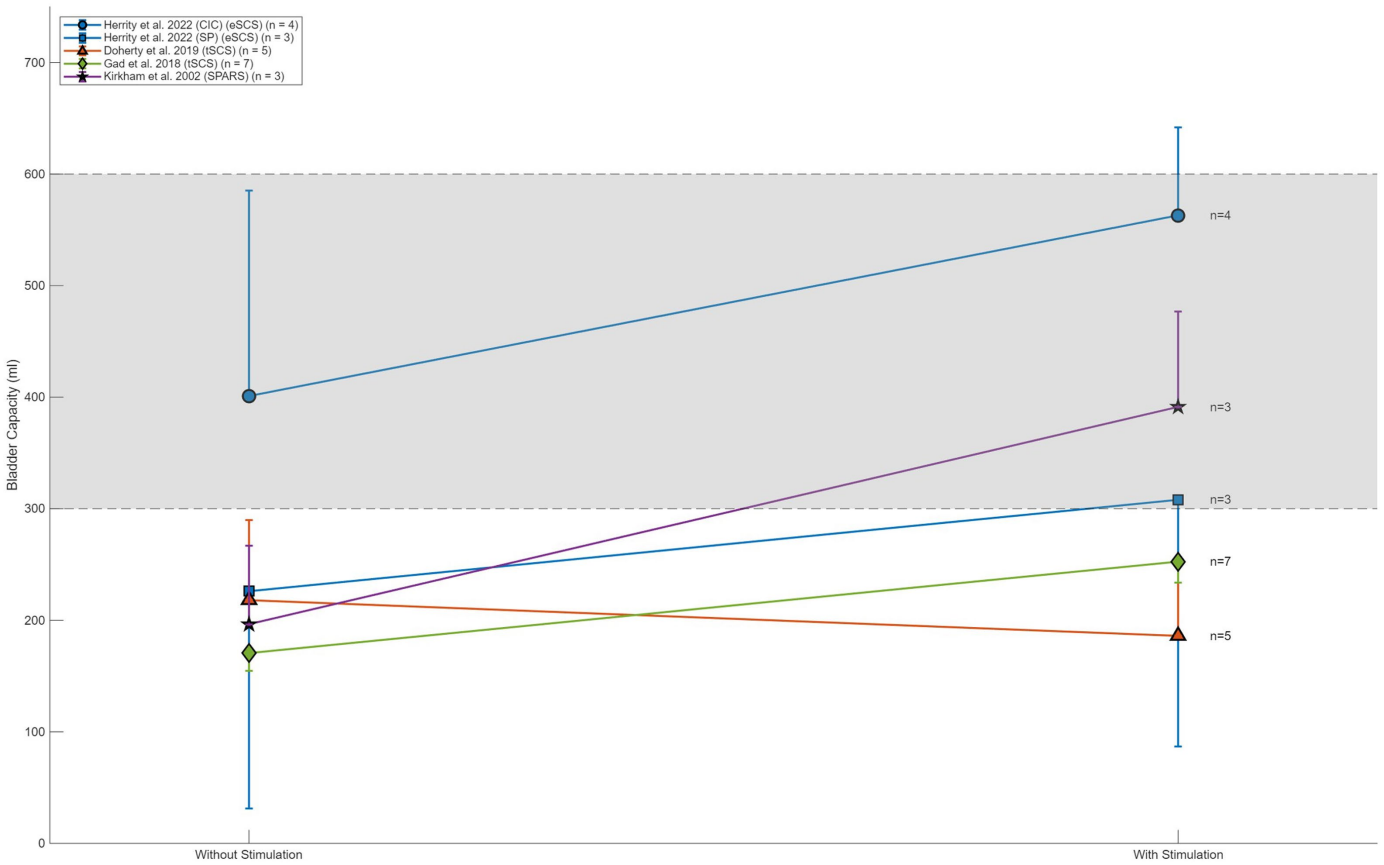
Mean (SD) bladder capacity (ml) without stimulation and with acute stimulation across studies using epidural (eSCS) or transcutaneous (tSCS) spinal cord stimulation or sacral posterior anterior root stimulation (SPARS). The grey area indicates the recommended bladder capacity (300–600 mL) from the International Continence Society guidelines. Sample sizes and modality of stimulation have been noted in the legend.

The acute effect of stimulation on bladder storage function during urodynamics, has been reported across one eSCS study (*n* = 7), two tSCS studies (*n* = 12) and one SARS studies (*n* = 3) (Table 6; Figure 4). All studies reported on bladder capacity (*n* = 17) or end fill volume (*n* = 5), and most reported on maximum detrusor pressure during storage (*n* = 12). Herrity et al. (2022) applied acute eSCS mapped to bladder function (*n* = 7). Mapping involved a minimum of 20 urodynamic sessions with parameter modification (anode/cathode positions, frequency and amplitude), noting that optimal configurations directed stimulation over the upper-mid lumbar segments (L1-L4). They demonstrated an acute increase in bladder capacity (+162 mL (*p* = 0.23), +82 mL (*p* = 0.54)) with a significant reduction in detrusor pressure (−50 cmH_2_0 (*p* = 0.001), −36 cmH_2_0 (*p* = 0.03)) across both intermittent self-catheterisation (ISC) (*n* = 4) and suprapubic catheter (SPC) (*n* = 3) groups, respectively. Of the two acute tSCS studies, one reported a significant increase in bladder capacity across 7 individuals (+82 mL (*p* < 0.05)) (Gad et al., 2018), whereas the other reported a non-significant reduction in end fill volume (−32 mL (*p* = 0.06)) alongside no change in detrusor pressure (*n* = 5) (Doherty et al., 2019). Both applied tSCS at T11-12 vertebrae, at frequencies of 15 Hz (Doherty et al., 2019) and 30 Hz (Gad et al., 2018). One trial, applying SPARS during urodynamic studies (*n* = 3) reported an acute increase in mean bladder capacity by 194 mL (Kirkham et al., 2002).

**Table 6:** Bladder capacity (ml), peak detrusor pressure (mmHg) and bladder compliance (ml/cmH_2_O) measured during urodynamics at baseline (without stimulation), during acute application of stimulation (stim) and post-intervention for chronic trials that applied stimulation using epidural (eSCS) or transcutaneous (tSCS) spinal cord stimulation or sacral anterior root stimulation (SARS) with sacral deafferentation (SARS-SDAF) or sacral posterior anterior root stimulation (SPARS).

Article	Stim type	Level targeted	Stim freq (Hz)	N	Capacity (ml)			Pdet (cmH_2_O)			Compliance (ml/cmH_2_O)		
					Baseline	Acute (with stim)	Chronic	Baseline	Acute (with stim)	Chronic	Baseline	Acute (with stim)	Chronic
Herrity et al. (2022)	eSCS (map)	T11-L1 Vertebral Target (L3-4)	60–85	4 (ISC)	401 ± 184	563 ± 79 (*p* = 0.2304)	NR	61 ± 33	14 ± 20 (*p* = 0.0007)	NR	NR		
				3 (SPC)	226 ± 195	308 ± 221 (*p* = 0.5385)	NR	64 ± 9	28 ± 5 (*p* = 0.0315)	NR	NR		
Herrity et al. (2021)	eSCS (map)	T11-L1 Vertebral (L1-S1 spinal)	NR	10	231 ± 134	NR	313 ± 166 *p* < 0.05 1 yr. FU: 324 ± 201 *p* < 0.05	53 ± 30	NR	29 ± 20 *p* < 0.01 1 yr. FU: 49 ± 20	9 ± 11	NR	8 ± 11
Beck et al. (2021)	eSCS	T12-L1 Vertebral (L1-S1 spinal)	NR	2	NR			NR			P1 = 62.5 P2 = 83.3	P1 = 12.5 P2 = 70	NR
Doherty et al. (2019)	tSCS	T11-12	15	5	EFV: 218 ± 72	EFV: 186 ± 69 (*p* = 0.063)	NR	83 ± 17	86 ± 21 (*p* = 1.00)	NR	NR		
Gad et al. (2018)	tSCS (map)	T11-12	30	7	170.54 ± 15.86	252.59 ± 18.91 mL (*p* < 0.05)	NR	NR			NR		
Krebs et al. (2017)	SARS-SDAF	S2-S5	NR	111	NR			17 (95%CI 15–19)	NR	7 (95%CI 4–13) (*p* = 0.1)	40 (95%CI 35–49)	NR	86 (95% CI 45–168) (*p* = 0.44)
Krasmik et al. (2014)	SARS-SDAF	S2-S5	NR	137	272.4 ± 143.0	NR	475.0 ± 82.7 (*p* < 0.001)	NR			NR		
Kirkham et al. (2002)	SPARS	S2-S4	15	3	196.5 ± 70.2*	391.3 ± 85.5*	NR	NR			NR		
Egon et al. (1998)	SARS-SDAF	S2-S5	NR	96	206	NR	564	NR			NR		
Van Kerrebroeck et al. (1997)	SARS-SDAF	S2-S5	NR	37	285.4 ± 172.0	NR	571.2 ± 141.9 (*p* < 0.001)	NR			27.4 ± 47.3	NR	48.5 ± 31.3 (*p* < 0.001)
Koldewijn et al. (1994)	SARS-SDAF	S2-S5	NR	27	228 ± 108	NR	5d post-op: 270 ± 104 6–12 m FU: 575 ± 122	NR			31 ± 22.1	NR	5d post-op: 14 ± 700 6-week FU: 44 ± 21.1 6–12-m FU: 48 ± 20.2

Spinal or vertebral level targeted with stimulation and stimulation parameters are also provided, with studies that performed mapping specifically for bladder function indicated (map). *Estimated using PlotDigitizer. CI, confidence interval; EFV, end fill volume; FU, follow-up; ISC, intermittent catheter; N, number; NR, not reported; P, participant; SPC, suprapubic catheter.

Two eSCS studies (*n* = 8), one tSCS study (*n* = 7), and one SARS studies (*n* = 6) investigated the acute effect of stimulation on voiding function (Table 7). In both eSCS studies, stimulation was optimised to target bladder function. Herrity et al. (2018) studied the effects of different configurations on voiding in a single participant. Stimulation of the lower lumbar sacral segments (L5-S1) using caudally positioned cathodes, at 30 Hz, produced the greatest effect, increasing voiding efficiency (VE) (8.3 ± 4.6% to 88.1 ± 1.1%) (Supplementary Table S2). Activation of the upper and middle portions of the array, corresponding to more rostral and mid-lumbosacral segments, yielded lower VE of 57.9 ± 21.1% and 68.5 ± 12.2%, respectively (Supplementary Table S2). These findings appeared to be participant-specific; when applied to four other participants, three of the four failed to achieve voiding efficiencies greater than 50%, and one demonstrated only 10% VE (Herrity et al., 2018). In a later study from the same group, voiding was achieved in seven individuals previously unable to void. VE data was however only reported for one participant, where VE increased from 0 to 51.2%. Across 7 participants, optimal configurations targeted the L4-S1 spinal level, at 20–35 Hz (Herrity et al., 2022). The one tSCS study (*n* = 7) evaluating voiding function demonstrated that tSCS at T11-12 vertebral level, at 1 Hz, increased VE from 27.0 ± 15.4% to 51.0 ± 5.3% (*p* < 0.05), PVR decreased from 183.9 ± 59.0 mL to 151.0 ± 54.1 mL (Gad et al., 2018). Within the study, they further compared T11-12 and L1-2 stimulation sites and observed stronger detrusor contractions with lower levels of urethral and abdominal activation at T11-12 compared to L1-2. The acute SARS trial noted a reduction in mean PVR from 161.6 ± 69.4 mL to 24.0 ± 5.5 mL for five of six participants, though one individual was unable to initiate voiding with stimulation (Arnold et al., 1986).

**Table 7:** Voiding efficiency (%) and post void residual (ml) measured during urodynamics at baseline (without stimulation), during acute application of stimulation (stim) and post-intervention for chronic trials that applied stimulation using epidural (eSCS) or transcutaneous (tSCS) spinal cord stimulation or sacral anterior root stimulation (SARS) with sacral deafferentation (SARS-SDAF) or sacral posterior anterior root stimulation (SPARS).

Article	Stim type	Level targeted	Stim freq (Hz)	*N*	Voiding efficiency (%)			Post void residual (ml)		
					Baseline	Acute (with stim)	Chronic	Baseline	Acute (with stim)	Chronic
Herrity et al. (2022)	eSCS (map)	T11-L1 Vertebral L4-S1 (Spinal)	20–35	1	0.0	51.2	NR	NR		
Herrity et al. (2021)	eSCS	T11-L1 Vertebral L1-S1 (Spinal)	NR	10	23 ± 27	NR	28 ± 33 1 yr. FU: 24 ± 24	NR		
Herrity et al. (2018)	eSCS (map)	T11-L1 Vertebral (Low Lumbar) Config A^+^	30	1	8.3 ± 4.6	88.1 ± 1.1	NR	NR		
	eSCS	T11-L1 Vertebral (Mid-Lumbar) Config B^+^		1	8.3 ± 4.6	57.9 ± 21.1				
	eSCS	T11-L1 Vertebral (Upper-Lumbar) Config C^+^		1	8.3 ± 4.6	68.5 ± 12.2				
Samejima et al. (2022)	tSCS	C3-C4, C6-C7, T11, L1	30 (10 kHz burst)	1	NR			125	NR	30
Gad et al. (2018)	tSCS	T11-12	1	7	26.99 ± 15.41	50.80 ± 5.25 (*p* < 0.05)	NR	183.86 ± 59.04	151.0 ± 54.12	NR
Schurch et al. (1997)	SARS-SDAF	S2-S5 (S3-S4 in 7/10)	NR	10	NR			157 ± 138	NR	16 ± 22
Van Kerrebroeck et al. (1997)	SARS-SDAF	S2-S5	NR	37	NR			104.7	NR	64.9
Arnold et al. (1986)^	SARS	S2-S5	NR	5	NR			161.7 ± 69.4	24.0 ± 5.5	NR

Spinal or vertebral level targeted with stimulation and stimulation parameters are also provided, with studies that performed mapping specifically for bladder function indicated (map). FU, follow-up; N, number; NR, not reported; P, participant. +For configs, see Supplementary Table S2. ^One participant could not void with stimulation, they are not represented in this table.

The effect of chronic stimulation has been assessed across two eSCS studies (*n* = 12) and one tSCS study (*n* = 2) for bladder function (Tables 6, 7). One study evaluating a chronic eSCS (L1-S1) and physical training intervention for locomotor function, reported significant increases in bladder capacity (+82 mL (*p* < 0.05)), compliance (+11 mL/cmH_2_0 (*p* < 0.01)) and reductions in detrusor pressure (−24 cm H_2_O (*p* < 0.01)) (*n* = 10) (Herrity et al., 2021). These parameters were also significantly elevated compared to a control-SCI cohort. At one-year follow-up, bladder capacity remained significantly increased (+93 mL (*p* < 0.05)), though detrusor pressure and compliance returned to baseline. There were no significant changes in VE post-intervention, nor at 1-year follow up, remaining at ~23% (Herrity et al., 2021). In contrast, another locomotor eSCS trial (*n* = 2) targeting similar spinal levels (L1-S1), reported unfavourable reductions in bladder compliance, leading to hyperreflexic bladder presentation in one individual (Beck et al., 2021). A chronic locomotor tSCS trial (*n* = 2), applying stimulation over 2 months, reported only on one of their participants, noting a reduction in PVR from 125 mL to 30 mL, facilitating discontinuation of ISC, which persisted 1.8 years on from study completion (Samejima et al., 2022).

Five chronic SARS-SDAF studies (*n* = 408) and one SARS study (*n* = 6) directly report on bladder storage parameters, with follow up ranging from 6 months to 14.8 years. Four of six trials reported on bladder capacity, all demonstrating improvements, ranging from +50 mL to +358 mL. The SARS trial that did not perform deafferentation of the posterior sacral nerve roots (Arnold et al., 1986), reported the smallest change in bladder capacity (+50 mL); which reduced during the follow-up period, though not back to baseline, whilst detrusor pressure decreased from 85.8 to 30.8cmH₂O. Only one SARS-SDAF trial (*n* = 111) reported on detrusor pressure, showing a reduction, 17 to 7cmH_2_0 at follow up (95%CI: (4, 13), *p* = 0.1) (Krebs et al., 2017). Three SARS-SDAF studies reported on bladder compliance, with values increasing by up to 50%, although non-significant (Koldewijn et al., 1994; Van Kerrebroeck et al., 1997; Krebs et al., 2017). Two SARS-SDAF studies (*n* = 53) applying chronic stimulation have captured voiding outcomes, with both reporting decreases in PVR by 39.8 mL (*n* = 37) (Van Kerrebroeck et al., 1997), and 141 mL (*n* = 10) (Schurch et al., 1997).

Ten SARS trials reported on qualitative LUT symptoms (*n* = 795). Nine out of 10 studies captured incontinence episodes (*n* = 728) and reported on incidence of UTI (*n* = 493) (Table 8), all noting improvements in incontinence episodes and UTI incidence. Two chronic locomotor eSCS trials (*n* = 4) report on incidence of UTI (Beck et al., 2021; Samejima et al., 2022). Beck et al. (2021) (*n* = 2) reported that though participants contracted a symptomatic UTI within 2 weeks of eSCS implantation, no further symptomatic UTIs were reported across an 18-month follow-up period. Samejima et al. (2022) (*n* = 2) reported that one of two participants experienced frequent UTI prior to eSCS—post-implantation there were no reported UTIs for up to 1.8 years. Incontinence was poorly reported within eSCS or tSCS trials, however, was captured within the NBSS subdomain in three chronic eSCS trials. One out of seven participants demonstrated a significant improvement in the incontinence subdomain, decreasing from 7 to 0 (Shackleton et al., 2023).

**Table 8:** Effects of chronic stimulation using epidural (eSCS) or transcutaneous (tSCS) spinal cord stimulation or sacral anterior root stimulation (SARS) with sacral deafferentation (SARS-SDAF) on subjective bladder outcomes: neurogenic bladder symptom score (NBSS), reported urinary incontinence episodes and urinary tract infection (UTI) burden.

Article	Stim type	Level targeted	Stim freq (Hz)	N	NBSS pre-intervention (baseline)	NBSS post-intervention
Samejima et al. (2022)	eSCS	multi-site C3-C4, C6-C7, T11, L1	30 (10 kHz burst)	2	Storage and Voiding: P1 = 7; P2 = 3 Incontinence: P1 = 8; P2 = 0; Consequences: P1 = 9; P2 = 4; Quality of Life: P1 = 3; P2 = 1	EOI: Storage and Voiding: P1 = 11; P2 = 5 Incontinence: P1 = 7; P2 = 0; Consequences: P1 = 5; P2 = 0; Quality of Life: P1 = 3; P2 = 2
						FU (2 months): Storage and Voiding: P1 = 7; P2 = 3 Incontinence: P1 = 0; P2 = 0; Consequences: P1 = 4; P2 = 0; Quality of Life: P1 = 2; P2 = 1
Darrow et al. (2019)	eSCS	T12 (Vertebral)	24–100 (P1) 30–50 (P2)	2	Storage and Voiding: P1 = 9; P2 = 3 Incontinence: P1 = 14; P2 = 7; Consequences: P1 = 4; P2 = 0; Quality of Life: P1 = 1; P2 = 2	Storage and Voiding: P1 = 5; P2 = 4 Incontinence: P1 = 13; P2 = 0; Consequences: P1 = 4; P2 = 0; Quality of Life: P1 = 2; P2 = 1
Shackleton et al. (2023)	eSCS	T11-12 (Vertebral)	NR	3	Storage and Voiding = 4.3 ± 4.2 Incontinence = 7.0 ± 7.0 Consequences = 2.7 ± 2.3 Quality of Life = 1.3 ± 0.6	Storage and Voiding = 4.7 ± 2.9 Incontinence = 4.3 ± 7.5 Consequences = 3.7 ± 3.2 Quality of Life = 1.3 ± 0.6
Beck et al. (2021)	eSCS	T12-L1 Vertebral L1-S1 (Spinal)	NR	2	P1 reported increase episodes of incontinence, whilst P2 with reduced occurrence of incontinence	Both participants contracted a symptomatic UTI within 2 weeks after implantation, however no further symptomatic UTIs were identified within both participants across the course of the 18 months intervention follow-up.
Zaer et al. (2018)	SARS-SDAF	S2-S4/5	NR	287	Significantly fewer episodes of incontinence (*p* < 0.0001)	NR
Castaño-Botero et al. (2016)	SARS-SDAF	S2-S4/5	NR	104	At baseline 100% of participants were incontinent, compared to only 14% after implantation (*p* < 0.001)	The urinary infection rate (defined as 1 or more severe UTIs a year) dropped from 95% of participants to 16% (*p* < 0.001)
Krasmik et al. (2014)	SARS-SDAF	S2-S4/5	NR	137	At baseline 60.9% of participants were incontinent, compared to only 38.3% after implantation (*p* < 0.001)	Mean number of symptomatic UTI per year decreased from 6.2 to 2.5 infections (*p* < 0.001).
van der Aa et al. (1999)	SARS-SDAF	S2-S4/5	NR	37	31 (84%) participants achieved complete continence. 5 (14%) participants had improvements in continence, and 1 (2%) patient was still incontinent	High incidence of UTI pre-operatively. Post-operatively UTI’s were rare.
Egon et al. (1998)	SARS-SDAF	S2-S4/5 (S3-S4 roots preferred)	NR	93	At baseline 92/93 (98.9%) individuals were incontinent, compared to only 11/93 (11.8%) surviving participants were incontinent	Pre-operatively all participants had chronic UTIs. Post-op 66/93 (70.9%) participants had resolution of chronic UTI with consistently negative cultures.
Schurch et al. (1997)	SARS-SDAF	S2-S4/5 (S3-S4 in 7/10)	NR	10	Preoperatively reflex incontinence was present in all participants (100%). Post-operatively reflex incontinence was eliminated in all participants (0%), although stress incontinence still persisted.	Chronic UTIs (three or more annually) decreased from 8 (80%) participants pre-op to 3 (30%) post-operatively
Van Kerrebroeck et al. (1997)	SARS-SDAF	S2-S4/5	NR	*n* = 52 (6 m FU *n* = 37)	Daytime continence rate increased from 10 to 73% of participants; Nighttime continence rate increased from 4 to 86% of participants.	Mean annual infections across cohort decreased from 1.94 to 0.38 per year.
Van Kerrebroeck et al. (1996)	SARS-SDAF	S2-S4	NR	52	Change in continence NR	In 44 participants followed up, mean incidence of UTI reduced from 4.2 (range 2–12) pre-op, to 1.4 (range 0–2), during last year of follow up.
van der Aa et al. (1995)	SARS-SDAF	S2-S4/5	NR	17	12/17 (70.6%) participants achieved complete continence	All participants had a high rate of UTI pre-operatively. This was significantly reduced post-operatively.
Arnold et al. (1986)	SARS	S2-S4/5	NR	6	At baseline 0% were completely continent. Post-operatively 5/6 (83.3%) participants achieved complete continence	High incidence of UTI pre-op which was then considerably reduced in 2/6 participants post implantation; another 2 of 6 participants had no infections

Spinal or vertebral level targeted with stimulation and stimulation parameters are also provided. EOI, end of intervention; FU, follow-up; N, number; NR, not reported; P, participant.

### 3.4 Bowel

Outcomes relating to bowel function are reported across 14 articles, using eSCS (*n* = 4), tSCS (*n* = 2), and/or SARS (*n* = 8), involving a total of *n* = 18 (eSCS); *n* = 6 (tSCS); and *n* = 649 (SARS) (Table 1). Across studies applying eSCS/tSCS, injury level ranged from C4-T12, with the majority having motor-complete injuries (AIS A–B), though one study applying tSCS included two individuals with incomplete cervical SCI (AIS D) (Samejima et al., 2022). In the context of SARS, participants’ injuries were mainly cervical or thoracic, with few lumbar injuries. Injuries were mainly all motor complete (AIS A-B), except for 18 individuals included across two studies with AIS C injuries (Egon et al., 1998, Krasmik et al., 2014). Most studies tracked effects of stimulation using bowel diaries throughout the span of a chronic stimulation intervention. Participants applied stimulation to directly facilitate BM in two eSCS studies (Walter et al., 2018; DiMarco et al., 2021) and all SARS studies. One tSCS study investigated the acute effect of tSCS on anorectal pressure profiles (Kreydin et al., 2022). The other studies involved BM without the presence of active stimulation (Darrow et al., 2019; Kreydin et al., 2022; Samejima et al., 2022) or with background sub-threshold stimulation (Kandhari et al., 2022a).

To date, no studies have undertaken systematic mapping of bowel networks. Stimulation has been primarily applied over the lumbosacral cord (L1-S1). Where electrode configurations for eSCS have been reported (Supplementary Table S2) this has typically been over the mid-caudal lumbosacral cord segments, i.e., L3 and below (Darrow et al., 2019; Kandhari et al., 2022a; Samejima et al., 2023a). Two studies applying SARS, report primarily targeting anterior roots S4/5 to improve bowel function (Van Kerrebroeck et al., 1996; van der Aa et al., 1999). In one study, stimulation applied at T9-T11, to enhance respiratory strength, also proved impactful for bowel function (DiMarco et al., 2021). Reported stimulation frequencies range from 24 to 60 Hz. One study that aimed to map the anorectal canal using tSCS, applied biphasic stimulation at 30 Hz with 10 kHz bursts. Pulse widths of 1 ms were utilised for tSCS and 210–450 μs for eSCS (Kreydin et al., 2022).

Outcome measures that have been adequately reported across the literature include BM time and BM frequency, measured via patient-recorded bowel diaries, and NBDS.

Seven studies including *n* = 17 (eSCS); *n* = 3 (tSCS) and *n* = 12 (SARS) participants have reported on the effect of stimulation upon BM time (Table 9). Only one case study explored the acute effects of stimulation, applying eSCS during bowel management, and reported a significant reduction in BM time with stimulation (Walter et al., 2018). Three configurations targeting the lumbosacral cord were compared (Supplementary Table S2), reducing BM time from a baseline of 58 ± 3 min to 23 ± 1 min (*p* < 0.05), 25 ± 3 min, and 31 ± 4 min (Walter et al., 2018). The configuration producing the greatest improvement (23 ± 1 min, *p* < 0.05) targeted right ankle dorsiflexion and right hip/knee flexion (L2–S1) (Walter et al., 2018). The other six studies assessing chronic stimulation (using eSCS, tSCS or SARS-SDAF), reported consistent improvements in BM time, with all six studies achieving completion of BM within 30 min (Table 9). Three out of six studies found a > 80% reduction in BM time (MacDonagh et al., 1990; DiMarco et al., 2021; Kreydin et al., 2022). Specifically, one case report demonstrated that 1 h of daily tSCS over 18 days led to a gradual reduction in BM time from 75 to 15 min in a single participant (Kreydin et al., 2022). Following this, sham tSCS was applied for another 18 days leading to a gradual increase in BM time up to 45–65 min, although not returning to baseline levels (Kreydin et al., 2022).

**Table 9:** Effects of chronic stimulation using epidural (eSCS) or transcutaneous (tSCS) spinal cord stimulation or sacral anterior root stimulation (SARS) with sacral deafferentation (SARS-SDAF) on bowel management (BM) time and frequency and neurogenic bowel dysfunction score (NBDS – severity subscale).

Article	Stim type	Level targeted	Stim freq (Hz)	N	BM time (mins)			BM frequency		NBDS score (severity)	
					Baseline	Acute (with stim)	Chronic	Baseline	Chronic	Baseline	Chronic
Shackleton et al. (2023)	eSCS	T11-12 (Vertebral)	NR	3	NR			NR		12.0 ± 3.6 (Moderate)	10.7 ± 2.9 (Moderate)
Kreydin et al. (2022)	tSCS (map)	T11-T12, L1-L2 (Cathode)	30 (10 kHz burst)	1	75	NR	15	NR		NR	
Kandhari et al. (2022a) and Kandhari et al. (2022b)	eSCS	T11-L1 vertebral region	60	10	25*	NR	17*	0.9/day*	1.15/day*	NR	
Samejima et al. (2022)	tSCS (map)	multi-site C3-C4, C6-C7, T11, L1	30 (10 kHz burst)	2	31–60	NR	EOI: P1: < 30; P2: < 30	P1: 7/weeks; P2: 2–3/weeks	EOI: P1: 7/weeks; P2: 6–7/weeks	P1 = 15 (Severe), P2 = 12 (Moderate)	EOI: P1 = 8 (Minor), P2 = 3 (Very Minor)
							FU (2 months) P1: 31–60; P2: <30		FU (2 m) P1: 7/weeks; P2: 6–7/week		FU (2 m) P1 = 11 (Moderate), P2 = 9 (Minor)
Darrow et al. (2019)	eSCS	T12 (Vertebral)	24–100 (P1) 30–50 (P2)	2	90	NR	<30	NR		P1 = 9 (Minor) P2 = 11 (Moderate)	P1 = 9 (Minor) P2 = 9 (Minor)
Walter et al. (2018)	eSCS	T11-L1 (Vertebral) Config A^+^	40	1	58 ± 3	25 ± 3	NR	NR		15 (Severe)	8 (Minor)
		T11-L1 (Vertebral) Config B^+^	30	1		23 ± 1 (*p* = 0.046)					
		T11-L1 (Vertebral) Config C^+^	40	1		31 ± 4					
DiMarco et al. (2021)	eSCS	T9-T11 spinal	50	5	118 ± 34	NR	18 ± 2	4.4 × per week	3.8 × per week	NR	
MacDonagh et al. (1990)	SARS-SDAF	S2-S5	NR	12	150.5	NR	28.9	5.5 × per week	8.3x per week	NR	
Rasmussen et al. (2015)	SARS	S2-S4	NR	277	NR			NR		17 (Median) (Severe)	11 (Median) (Moderate)

*Estimated using PlotDigitizer. ^+^For Configs, refer to Supplementary Table S2. Spinal or vertebral level targeted with stimulation and stimulation parameters are also provided., with studies that performed mapping specifically for bladder function indicated (map). EOI, End of intervention; FU, follow-up; N, number; NR, Not reported; P, participant.

Bowel management frequency has been reported across four studies evaluating chronic stimulation, including *n* = 15 (eSCS), *n* = 2 (tSCS) and *n* = 12 (SARS-SDAF) participants (Table 9). Most participants had a baseline BM frequency of between 5 and 7/week. In three out of four studies, BM frequency was either maintained at, or increased, towards daily BM frequency (7/week). One study applying combined cervical (C3-C6 and C6-7 vertebrae) and lumbosacral (T11-L1 vertebrae) tSCS within two participants, reported that one of their participants had an increase from BM 2–3/week up to 6–7/week, whilst the other maintained a frequency of 7/week (Samejima et al., 2022). Similarly, one study applying SARS (S2-S5), reported an improvement in mean BM frequency across 12 participants from 5.5 to 8.3 times a week (MacDonagh et al., 1990). Only one study applying chronic eSCS at T9-T11 to improve respiratory strength showed a modest decrease in BM frequency—importantly, they also report a significant improvement in BM time (*p* < 0.05) (DiMarco et al., 2021).

The NBDS has been reported across 5 studies evaluating chronic stimulation interventions, including *n* = 6 (eSCS), *n* = 2 (tSCS), and *n* = 277 (SARS-SDAF) participants (Table 9). Of those applying SCS, four out of eight participants reported a reduction in severity grade (e.g., severe to moderate or moderate to mild) (Walter et al., 2018; Darrow et al., 2019; Samejima et al., 2022), with the other 4 participants showing little to no change in NBDS (Darrow et al., 2019; Shackleton et al., 2023). After completion of 2 months of daily tSCS and locomotor training, two participants reported reductions in their NBDS score and severity from 15 (Severe) and 12 (Moderate), to 8 (Minor) and 3 (Very Minor), respectively (Samejima et al., 2022). At follow up, 2 months after the completion of intervention, NBDS scores remained reduced compared to baseline with scores of 11 (Moderate) and 9 (Minor), respectively (Samejima et al., 2022). Darrow et al. (2019) reported a temporary worsening of NBDS grade across both participants from mild to moderate in one, and moderate to severe in the other during one of their follow-up visits—although by the end of the follow-up period NBDS returned to baseline in one participant and improved in the other (Darrow et al., 2019). A large case-series showed that SARS led to a reduction in median NBDS score from 17 (severe) to 11 (moderate) across 277 individuals (*p* < 0.0001) (Rasmussen et al., 2015).

### 3.5 Sexual function

Outcomes relating to sexual function were reported in 13 articles using eSCS (*n* = 4), and/or SARS (*n* = 9), involving a total of *n* = 17 (eSCS) and *n* = 646 (SARS/SARS-SDAF) participants (Table 1). tSCS has not yet been studied for sexual function. Across studies applying SCS, injury level ranged from T2-T12 with all injuries being AIS A. In the context of SARS, participants injuries were mainly cervical or thoracic, with few lumbar injuries. Injuries were mainly all complete (AIS A/B), except for 18 individuals included across two studies (Egon et al., 1998, Krasmik et al., 2014). Outcomes related to sexual function were typically evaluated after chronic interventions via questionnaires or follow-up appointments. When eSCS was applied, stimulation targeted the L1-S1 lumbosacral roots for locomotor function. To date, only one eSCS study has aimed to directly target sexual function utilising a 32-electrode array (Supplementary Table S2), to target the L4 spinal segments in 2 males (Rybka et al., 2024). Here, stimulation was applied as a 20 Hz monophasic waveform with a pulse width of 210 μs. Frequencies utilised across other papers, where reported, range between 15 and 100 Hz (Darrow et al., 2019; Kandhari et al., 2022a). With regards to SARS, where individual root stimulation to facilitate erection was reported, most studies utilised S2 anterior root stimulation (*n* = 104) (Arnold et al., 1986; van der Aa et al., 1995; Van Kerrebroeck et al., 1996; Van Kerrebroeck et al., 1997; Egon et al., 1998; van der Aa et al., 1999), with few reporting sole S3 stimulation (*n* = 31) (van der Aa et al., 1995; Egon et al., 1998; van der Aa et al., 1999).

Outcomes adequately reported in males pertain to presence of erectile (arousal) and ejaculation (orgasm) function. Twelve studies (*n* = 12 (eSCS) and *n* = 589 (SARS)) report outcomes relating to Erectile function (Table 10). Of the two eSCS studies (*n* = 12), one study reports that 2/2 participants were able to achieve erection (Rybka et al., 2024). The other study reported that with subthreshold broad-field chronic eSCS, 5/10 patients had improvements in erectile function, both reflex and psychogenic (Kandhari et al., 2022a). In the context of SARS, deafferentation (SDAF) invariably leads to a loss of erectile function. In the two studies (*n* = 11) where SDAF was not performed, across 8 male patients, 7 had preserved erections – the quality of erections was maintained across 6 out of these 7 patients, who were able to use this for sexual intercourse (Arnold et al., 1986; Kirkham et al., 2002). Across the eight studies (*n* = 579 males) utilising SDAF, erection is driven by anterior root stimulation. Sustained full erection driven by continuous stimulation was possible in most cases. However, a large cohort case-series showed that only 17.5% (27 out of 154) found SARS sufficient for sexual intercourse (Zaer et al., 2018).

**Table 10:** Effects of chronic stimulation using epidural spinal cord stimulation (eSCS) or sacral anterior root stimulation (SARS) with sacral deafferentation (SARS-SDAF) on sexual function: number (N) of participants that achieved erection or ejaculatory reflex.

Article	Stim type	Level targeted	Stim frequency (Hz)	*N*	Erection achieved/improved (N)	Ejaculatory reflex achieved (N)
Kirkham et al. (2002)	SARS	S2-S4 (spinal)	15	5	5	NR
Egon et al. (1998)	SARS-SDAF	S2-S4 (spinal) S2 (*n* = 34) S3 (*n* = 2) S2 + S3 (*n* = 10)	NR	65	46	NR
Van der Aa et al. (1999)	SARS-SDAF	S2/S3 (spinal) S2 (*n* = 27) S3 (*n* = 2)	NR	33	29	NR
Rybka et al. (2024)^	eSCS (map)	L1-S1 (spinal)P1: Rostral part of array (hip and knee extension) P2: targeting L4	70 (top of array); 20 (bottom of array)	2	2	2
Kandhari et al. (2022a) and Kandhari et al. (2022b)	eSCS	T11-L1 (vertebral) mapped for locomotor training	15–60	10	5*	0*

Spinal or vertebral level targeted with stimulation and stimulation parameters are also provided, with studies that performed mapping specifically for sexual function indicated (map). NR, not reported; P, participant. *Estimated using PlotDigitizer; ^^^Rybka et al. (2024) measured the acute effects of stimulation.

Three studies (*n* = 12 (eSCS) and *n* = 154 (SARS)) report outcomes relating to ejaculation (Table 10). In the one study where eSCS was applied to target sexual function, 2/2 participants achieved ejaculation (Rybka et al., 2024). Of significant note, one of the two participants was also able to achieve natural conception through sexual intercourse (Rybka et al., 2024). In the other eSCS study, none of the patients in the cohort achieved ejaculation (Kandhari et al., 2022a). A large retrospective case series, evaluating SARS-SDAF reported that pre-operatively 49/154 patients were capable of ejaculation. Following SARS-SDAF, this reduced to 10/154 (*p* < 0.001) (Zaer et al., 2018).

Only four studies (*n* = 5 eSCS; *n* = 136 SARS) assessed female arousal and orgasm function (Darrow et al., 2019; Shackleton et al., 2023; Zaer et al., 2018; Arnold et al., 1986), but the heterogeneous outcome measures employed, precluded further analysis of female sexual function.

### 3.6 Respiratory function and thermoregulation

Outcomes related to respiratory function were reported in 14 articles using eSCS (*n* = 12) and/or tSCS (*n* = 3), involving a total of *n* = 53 (eSCS) or *n* = 14 (tSCS) participants (Table 1). Primarily the thoracolumbar region was targeted (T9-L1 segments) (DiMarco et al., 2006; DiMarco et al., 2009; DiMarco et al., 2014; DiMarco et al., 2018; DiMarco et al., 2019; DiMarco et al., 2020; DiMarco et al., 2021; Kumru et al., 2023); although some targeted cervical segments (C3-C6) (Gad et al., 2020; Kumru et al., 2023), higher thoracic segments (T1-T5) (DiMarco et al., 1994; DiMarco et al., 2005; Gad et al., 2020; Kandhari et al., 2022b), or lumbosacral roots (Nightingale et al., 2019; Hodgkiss et al., 2024). The majority of eSCS studies (nine of twelve) were reported by the DiMarco group. Reported outcome measures include Maximum Inspired Volume, Maximal Expiratory Airway Pressure, and Peak Expiratory Flow. Since these measures primarily reflect somatic motor control and respiratory muscle strength, rather than autonomic function, we decided to exclude respiratory function from further analysis.

We were unable to identify any studies that systematically examined or reported quantitative outcomes related to thermoregulation within SCI patients.

## 4 Discussion

In this review, we have collated the published literature on the effects of direct stimulation of the spinal cord and/or roots on autonomic function within complete and incomplete SCI. Our main finding was that acute eSCS or tSCS (applied invasively or non-invasively) targeting the lumbosacral nerve roots reliably elevated blood pressure during hypotensive states. Acute effects on bladder, bowel, and sexual function were more variable, though targeted mapping was conducted in very few cases. Stimulation of the sacral anterior roots (SARS) consistently improved storage and voiding when deafferentation of the posterior sacral nerve roots was performed. We also found some evidence that acute stimulation of the lumbosacral nerve roots may improve bladder capacity, but these effects were reported less consistently. Other than large retrospective trials of SARS, few studies investigated the chronic effects of spinal cord or nerve root stimulation on autonomic function.

### 4.1 Cardiovascular neuromodulation

Across the 22 studies reviewed, acute SCS elicited robust pressor responses at rest and with positional hypotension (Figures 2, 3). These findings were clinically meaningful, with seven of eight studies (*n* = 15) reporting that individuals no longer met the clinical criteria for orthostatic hypotension, and in cases allowing clinical resolution of orthostatic-induced symptoms, e.g., light-headedness, dizziness, poor concentration and improvements in cognitive function (Phillips et al., 2018; West et al., 2018; Darrow et al., 2019). Most studies included in the review applied acute SCS targeting the lumbosacral enlargement, though minor increases in BP have been noted with cervical or upper thoracic stimulation in one study (Engel-Haber et al., 2024). The sympathetic circuitry underlying hemodynamic regulation, namely the Sympathetic Preganglionic Neurons (SPNs), are located across the thoracolumbar cord (T1-L2). In a rodent model, Squair et al. (2021) reported that the highest density of SPNs lies within the caudal thoracic spinal segments (T10-T12), which they labelled the ‘haemodynamic hotspot’. When stimulation was applied targeting T10-T12 segments, it produced robust corrections in BP during orthostatic stress in rodents (Squair et al., 2021) and in 14 humans (Phillips et al., 2025). Lumbosacral SCS may also indirectly engage the SPNs at this ‘hotspot’ by targeting mid-upper lumbar roots combined with electric field spread to caudal thoracic segments (Capogrosso et al., 2013; Squair et al., 2021). However, the caudal thoracic ‘hotspot’ hypothesis is largely based on complete-transection animal models of SCI, which may not reflect the heterogeneous pathology in human SCI. An alternative explanation is that lumbosacral SCS directly activates the somato-autonomic reflex to trigger pressor responses – recruitment of large-diameter dorsal-root afferent fibres (Ladenbauer et al., 2010), may excite ascending spinal interneurons (Conta and Stelzner, 2004) that project to SPNs producing splanchnic vasoconstriction and pressor responses.

The effects of acute SCS on suppressing hypertensive episodes (AD) were much less consistent, with reported suppression, no effect, or exacerbation of response across studies. It is perhaps unsurprising that SCS cannot selectively excite SPNs in hypotensive cases and inhibit them during AD (Solinsky et al., 2024). In healthy individuals, the somato-autonomic reflex is modulated via supraspinal input (Wulf and Tom, 2023). However, with the disruption to central regulation in SCI, such context-dependent modulation with SCS in this population seems unlikely. Even so, others have proposed that SCS modulates the excitability of spinal autonomic circuits (West et al., 2018), enhancing residual supraspinal input, allowing more physiological adaptive autonomic responses. Consistent with this, SCS-induced haemodynamic modulation has been reported to only occur in people with baseline instability, whereas those with more intact function—either SCI individuals without dysregulation, or healthy controls—showed minimal responses to stimulation and maintained normotensive BP (Aslan et al., 2018; Solinsky et al., 2024).

Two studies in our review report that tSCS-evoked increases in resting BP which were accompanied by significant reductions in HR, raising concern that pressor responses may reflect low-level AD (Engel-Haber et al., 2024; Hodgkiss et al., 2024). Indeed, tSCS further exacerbated the response to noxious stimuli (Solinsky et al., 2024), and chronic lumbosacral eSCS has been reported to approximately double the magnitude of SBP elevation and severity of AD in mice (Soriano et al., 2025). A recent pre-clinical study identified aberrant projections from the lumbosacral cord to thoracic sympathetic networks as key drivers of the AD response (Soriano et al., 2025). Therefore, it is possible that lumbosacral stimulation recruits the same maladaptive pathways implicated in AD. One group applying tSCS over the caudal thoracic cord (T7-8 vertebrae), found attenuation of AD and stabilisation against orthostatic stress (Phillips et al., 2018; Sachdeva et al., 2021), suggesting it might be a safer target. Further research is needed to determine the safety of pressor responses elicited by thoracolumbar or lumbosacral SCS and to distinguish these effects from pathological AD.

Evidence surrounding heart rate and blood pressure variability (HRV and BPV), remains sparse (*n* = 10). Preliminary data suggest that acute SCS may partially restore the blunted variability observed after SCI. Increases in total HRV power, particularly within the LF bands, during orthostatic stress, may reflect greater autonomic engagement (Legg Ditterline et al., 2021; Gorgey et al., 2023). The LF and HF components of HRV are thought to represent baroreflex mediated sympathetic activity and parasympathetic drive, respectively (Shaffer and Ginsberg, 2017). However, these interpretations have been controversial, given confounding influences such as respiratory frequency. Notably, only one study controlled for this using pulmonary pacing (Solinsky et al., 2024), limiting reliability and comparability of these findings. Moreover, HRV gains during orthostatic stress were not paralleled by LF BPV changes, leaving the extent of sympathetic vasomotor involvement uncertain. Findings during AD challenges were also inconsistent (Samejima et al., 2023a; Solinsky et al., 2024). Overall, a broader assessment of autonomic parameters is essential to clarify the underlying physiological effects. Although changes in BP and HR are frequently reported, these measures alone cannot distinguish whether SCS restores integrated autonomic regulation or simply induces acute pressor responses. Incorporating additional surrogate markers—such as spectral components of HRV and BPV, thought to represent, as well as serum catecholamines—will be important to probe this distinction.

Recent preclinical studies demonstrate that chronic eSCS drives adaptive remodelling, augmenting both aberrant and functionally beneficial projections, preventing haemodynamic instability (Soriano et al., 2025). Within our review, only four studies evaluated chronic stimulation for cardiovascular outcomes, with three showing signs of adaptive change. One case series (*n* = 4) report that BP was stable during orthostatic stress, even in the absence of stimulation, and was also associated with improvements in cardiac morphology and function (Legg Ditterline et al., 2020). Another case report found that, although the magnitude of orthostatic drop remained unchanged post-intervention (without eSCS), OH could be corrected with lower stimulation intensities (Gorgey et al., 2023). Early work by Richardson et al. (1979) showed that daily lumbosacral eSCS led to complete resolution of AD over 2 years. In contrast, Herrity et al. (2021) found no improvement in AD severity after 160 sessions of stimulation combined with rehabilitation—though stimulation was mapped for locomotion rather than cardiovascular control.

Out of 22 SCS studies, only 7 performed optimisation to directly target CVS function. Most studies applied stimulation at frequencies between 15 and 60 Hz directed towards the thoracolumbar spine (T10-L2) (Supplementary Table S2) either determined by CVS mapping or for targeting lower limb muscles. Only one study directly compared stimulation frequencies and reported the greatest sympathetic activation with tSCS at 120 Hz compared to 30 Hz (with or without 5 kHz bursts) (Solinsky et al., 2024). Regarding waveform, most studies applied monophasic pulses, though Sachdeva et al. (2021) suggested that biphasic tSCS pulses were preferable to suppress AD. However, one other study reported worsening AD with biphasic stimulation (Solinsky et al., 2024). No studies directly compared different pulse widths on cardiovascular outcomes; however, a recent review that examined stimulation parameters in applications beyond SCI, reported that shorter pulse widths (100–200 μs) decreased BP whereas longer pulse widths (500–600 μs) increased BP (Law et al., 2024). Moving forward, systematic comparisons of stimulation parameters and standardised mapping strategies for optimisation are required.

### 4.2 Bladder

Acute SCS demonstrates variable effects on lower urinary tract (LUT) function, with positive outcomes heavily dependent on bladder mapping (Table 6). In two studies, targeted eSCS or tSCS improved storage dynamics by suppressing NDO, increasing compliance, and maintaining low filling pressures, collectively supporting increases in bladder capacity (Gad et al., 2018; Herrity et al., 2022). Furthermore, in the only study that directly compared the effectiveness of eSCS with a cohort of SCI controls, bladder capacity, detrusor pressures, and bladder compliance were similar between groups at baseline. Following eSCS, however, all three storage parameters improved significantly relative to controls. Despite the noted improvements in bladder capacity with stimulation, translation to clinically desirable ranges have been limited: amongst 22 individuals with suboptimal baseline capacities, only nine reached the ICS-recommended 300–600 mL threshold (Rosier et al., 2013) (Figure 4). Acute SPARS (without rhizotomy) notably led to a doubling of bladder capacity in two out of three individuals (Kirkham et al., 2002). Evidence on voiding outcomes with acute eSCS or tSCS remains limited, though all three studies included, consistently report enhanced voiding efficiencies (Gad et al., 2018; Herrity et al., 2018; Herrity et al., 2022). Despite these improvements, two of these studies still report suboptimal bladder emptying with VE of ~ 50%. Where SARS is applied, acute stimulation of the anterior roots is shown to directly drive stronger detrusor contractions and facilitate emptying – however in the absence of rhizotomy and with persisting DSD, it could worsen dyssynergia and conversely impair voiding function (Kirkham et al., 2002).

Neural control of the LUT depends on coordinated supraspinal and spinal circuits, namely the Pontine Micturition Centre (PMC) and the spinal somatic (Onuf’s nucleus, S2–S4), sympathetic (T10-L2), and parasympathetic pathways (S2–S4) (Fowler et al., 2008). During bladder filling, pelvic afferent input activates spinal reflexes, contracting the urethral sphincter and suppressing detrusor activity via somatic and sympathetic efferents, respectively. Voiding is mediated through parasympathetic activation (S2-S4), contracting the detrusor and inhibiting sympathetic and somatic outflow, thereby relaxing the sphincter (Fowler et al., 2008). Targeted modulation of these spinal networks may hence offer a mechanistic basis for neuromodulatory interventions.

In the context of storage function, one study applying SPARS (S2–S4) (Kirkham et al., 2002) reported increases in bladder capacity likely by engaging the physiological continence pathways described above. Peripheral neuromodulatory techniques such as DGNS and SNM similarly improve storage function by recruiting these pudendal sacral afferents. In contrast, eSCS—and particularly tSCS—can engage lumbosacral roots with far less selectivity than SARS. Whilst SCS has been shown to reliably activate posterior roots related to lower limb muscles, there is only some evidence indicating that it can selectively recruit the dorsal roots involved in bladder and sphincter control (Jantz et al., 2022). Inadvertent activation of the parasympathetic efferents, rather than or alongside afferent activation, during filling, could provoke detrusor contractions and worsen storage function. Indeed, two studies note adverse effects of eSCS and tSCS in a subset of individuals, reporting worsening bladder capacity and exacerbated detrusor hyperreflexia (Doherty et al., 2019; Beck et al., 2021). Where safe and meaningful improvements in bladder capacity with eSCS have been reported, stimulation has instead targeted the upper-mid lumbar segments (L1-L4) (Herrity et al., 2022), suggesting that activation of thoracolumbar sympathetic networks may surmise a more effective strategy to enhance bladder storage. Improvements in voiding function have largely been noted where more caudal lumbosacral roots are targeted, particularly in the context of SARS through activation of parasympathetic efferents (S2-S4). Synchronous contraction of the urethral sphincter, via adjacent somatic efferents (S2-S4), is counteracted using burst stimulation paradigms, which permit voiding in pulses by exploiting the detrusor’s longer relaxation time relative to the sphincter (Arnold et al., 1986). eSCS targeted for voiding function has also been applied over more caudal lumbosacral segments (L4-S1) (Herrity et al., 2018, 2022), possibly also modulating the parasympathetic networks. Preclinical research has further identified a lumbar spinal coordinating centre (L3–L4) with rhythmic bursting activity in rodent models of SCI, that plays a critical role in detrusor and sphincter coordination across storage and voiding phases in rats (Abud et al., 2015). Although the translational relevance of this coordinating network in humans remains to be established, its presence highlights a further target for neuromodulation of the LUT. Furthermore, beyond direct activation of the segmental spinal circuits, SCS may also facilitate the integration of residual descending supraspinal input by increasing the excitability of spinal networks (Steadman and Grill, 2020; Herrity et al., 2021). Herrity et al. (2022) leveraged this principle through ‘patient-driven’ mapping approaches, utilising sensory feedback and patient intent, to guide optimisation of stimulation parameters.

The effects of chronic stimulation on bladder capacity or voiding were only investigated in one eSCS study, reporting a significant improvement after 160 sessions of eSCS combined with locomotor training (*n* = 10). Others have reported the effects of chronic SCS interventions on qualitative outcomes, such as the Neurogenic Bladder Symptom Score (NBSS); however, little to no changes were reported outside of the incontinence domain. Some studies report individual functional gains including reduced reliance on intermittent catheterisation, decreased incontinence episodes, improved sensation of bladder fullness, and lower urinary tract infection burden (Beck et al., 2021; Kandhari et al., 2022a; Samejima et al., 2022; Herrity et al., 2022). Whilst much more research is needed, chronic SCS may drive adaptive remodelling, reducing maladaptive C-fibres afferents that contribute to detrusor overactivity (De Groat, 1995). Most chronic SCS studies in our review evaluated long-term outcomes in SARS–SDAF cohorts. These cohorts consistently demonstrated large improvements in storage function: bladder capacity roughly doubled (e.g., 285 mL to 571 mL) compliance increased (27.4 to 48.5 mL/cmH₂O), and storage pressures declined (Table 6). Contemporary series also report major reductions in incontinence (100 to 14%) and symptomatic UTIs (95 to 16%) (Castaño-Botero et al., 2016) (Table 8). However, these marked improvements likely reflect the effects of rhizotomy, known to produce an areflexic, highly compliant bladder, rather than neuromodulatory action of SARS.

In terms of stimulation parameters, SARS parameters were seldom reported but would be limited to the device capabilities (pulse width 24–720 μs and frequency 8–46 Hz) and SCS was typically applied between 15–85 Hz. Where mapping was undertaken, application of ‘higher’ frequencies (30–85 Hz) favoured low-pressure storage, whilst lower frequencies facilitated voiding (1–35 Hz). Adaptive systems capable of providing phase-specific stimulation may therefore yield superior outcomes in the future (Hokanson et al., 2021), mirroring phase-switching normally mediated by the PMC (Fowler et al., 2008). Moreover, it would be beneficial to test and compare a range of stimulation paradigms. For example, burst stimulation paradigms may mimic physiological bursting patterns such as that of the lumbar coordinating centre (Abud et al., 2015).

### 4.3 Bowel

All modalities (eSCS, tSCS, and SARS) consistently shortened bowel-management (BM) duration, with most individuals achieving programme times < 30 min. Baseline BM frequency in all reported cohorts already met recommended minimums (≥3/week) and generally remained stable or improved slightly, with most individuals reaching near-daily BMs (6–7/week). Several studies also reported improved bowel sensation, reduced constipation and incontinence rates (MacDonagh et al., 1990; Vallès et al., 2009; Kandhari et al., 2022a; DiMarco et al., 2021; Samejima et al., 2022). Collectively, these findings suggest that acute and chronic SCS may partially overcome prolonged colonic transit and dyssynergic defecation inherent to SCI.

The GI tract is innervated by the enteric nervous system (ENS), specifically the Auerbach’s and Meissner plexus. These networks are however modulated by supraspinal control through the spinal somatic (S2-S4), sympathetic (T10-L3), and parasympathetic (CN X and S2-S4) control (Rodriguez and Gater, 2022). Together, these directly control and coordinate peristalsis, mucosal secretion, and defecation. During suprasacral SCI, although the ENS is intact, the loss of supraspinal modulation, results in overactive, but poorly coordinated, reflex activity leading to impaired colonic propulsion, sphincter dyssynergia and ultimately constipation (Rodriguez and Gater, 2022). Emerging evidence suggests that SCS can modulate these dysfunctional reflexes. Acute tSCS has been shown to alter anorectal pressure profiles, with maximal responses, 0.5–3 cm above the anal verge, approximating to the external anal sphincter (EAS) (Kreydin et al., 2022). This suggests that somatic pudendal circuits, innervating the EAS, are the final element of neuromodulation. However, prolonged response latencies point to a polysynaptic pathway rather than direct nerve activation, consistent with modulation of broader interspinal networks (Kreydin et al., 2022). Reviewing stimulation strategies, we note that one eSCS study (DiMarco et al., 2021) and all SARS studies employ intermittent SCS, delivered in periodic on/off cycles of seconds to minutes. Preclinical data indicate that intermittent SNM more effectively facilitates colonic motility than tonic stimulation, likely by aligning with intrinsic rhythmicity of colonic propulsive contractions (Barth and Grill, 2025). Paradigms better matched to physiological activity may therefore yield superior outcomes and warrant further study.

Several studies reported bowel improvements using electrode configurations targeting mid-caudal lumbosacral segments, i.e., L3 and below (Darrow et al., 2019; Kandhari et al., 2022a; Samejima et al., 2023a), suggesting preferential targeting of parasympathetic circuits that support motility and defecation function. eSCS over T9-T11, targeting cough function, also significantly reduced bowel programme times, likely via the increased intra-abdominal pressures generated rather than autonomic modulation—however further investigations are required (DiMarco et al., 2021). Significant improvements in bowel function with SARS-SDAF also highlight sacral roots, particularly S4/S5, as key targets, modulating both parasympathetic and somatic-driven external anal sphincter tone. Preclinical data, however, highlights the complexity of this modulation. In rodents, SCS applied at T13-L2 levels inhibited rectal contractions, consistent with activation of sympathetic fibres, however, simultaneously also increased distal colonic motility. Conversely, stimulation at the L5-S1 segments, expected to enhance parasympathetic output, instead suppressed rectal contractions (Hoey et al., 2021; Hoey et al., 2022). These results suggest that lumbosacral SCS may instead activate integrated spinal circuits whose functional output cannot be predicted by sympathetic vs. parasympathetic characterisations alone. SCS may instead increase the overall excitability of cord, facilitating communication with ENS, restoring greater physiological tone.

Changes in NBDS with eSCS and tSCS are variable, with half of the participants reviewed reporting decreases in severity grade, and the other half reporting no change. Notably, one study (*n* = 2) did report that improvements in NBDS persisted at follow-up after completion of a chronic intervention (Samejima et al., 2022). Qualitative improvements with SARS-SDAF have been more robust, decreasing median NBDS score from 17 (severe) to 11 (moderate) across a cohort 277 individuals (*p* < 0.0001) (Rasmussen et al., 2015). Key longitudinal findings beyond those reported within our review include greater independence, reduced reliance on bowel aids (e.g., digital evacuation, suppositories, enemas), and use of SARS as the primary method of bowel emptying across 67% of a the cohort (Castaño-Botero et al., 2016; Rasmussen et al., 2015).

To date, no studies have directly mapped for bowel function. Future research must address this gap through rigorous mapping of gastrointestinal responses—further examining colonic transit time, anorectal pressure dynamics, and sphincter activity (Samejima et al., 2023b). Developing reproducible protocols for bowel-specific neuromodulation will be essential if SCS is to move from incidental improvements to targeted therapy in this domain.

### 4.4 Sexual

Sexual outcomes after SCS remain infrequently reported, including only four eSCS studies (*n* = 17) and select SARS cohorts. Early findings in males remain promising, where both acute eSCS and SARS (without deafferentation) appear to preserve or enhance erectile function (Arnold et al., 1986; Kirkham et al., 2002; Rybka et al., 2024). Kandhari et al. (2022a) reported that chronic subthreshold lumbosacral eSCS led to improvement of psychogenic and reflexogenic erection, though ejaculation was not achieved. Notably, in one recent study, application of targeted acute eSCS facilitated both erection and ejaculation in response to penile vibratory stimulation, in two participants (Rybka et al., 2024). Furthermore, chronic eSCS also enabled one of the participants to achieve ejaculation by masturbation and allowed successful natural conception—an isolated but important result demonstrating the potential of this technology.

Findings pertaining to the impact of eSCS on female sexual health, however, remain extremely limited with only two studies reporting on outcomes (Darrow et al., 2019; Shackleton et al., 2023). This likely reflects the demographic distribution of SCI, alongside the underrepresentation of women in SCI research. Where reported, standardised questionnaires, such as the Female Sexual Function Index (FSFI) and Female Sexual Distress Scale (FSDS) (Rosen et al., 2000; Derogatis et al., 2002), reflect modest improvements in the arousal domains (psychogenic), but minor drops in the lubrication domains (Shackleton et al., 2023). Improvements in orgasm function were more consistent across both studies, specifically with consistent restoration of orgasm in one participant with active stimulation (Darrow et al., 2019; Shackleton et al., 2023). These results highlight the capacity of SCS not only to restore sexual function but also to improve subjective sexual experience and satisfaction.

Like other pelvic organs, sexual function is also controlled by sympathetic (T10–L2), parasympathetic (S2-S4) and somatic circuits (S2-S4) circuits throughout the thoracolumbar and lumbosacral cord. SCS may enhance sexual function by facilitating thoracolumbar sympathetic outflow (T10–L2), restoring psychogenic arousal (Krassioukov and Elliott, 2017). Additionally, activation of sacral parasympathetic and somatic circuits (S2–S4) can drive reflexogenic genital responses. SCS may also increase the excitability of spinal networks facilitating ascending sensory transmission, and integrate residual supraspinal input, improving psychogenic arousal and orgasm. Recently, Rybka et al. (2024) directly targeted the Spinal Ejaculation Centre (SEG) a well-characterised interneuronal network within the L3-L5 spinal segments, known to coordinate the sympathetic, parasympathetic and pudendal neurons involved in ejaculation function (Chéhensse et al., 2017). Targeting the L4 spinal segments led to noticeable restoration of ejaculation function in two participants, highlighting the possible specificity of this region in enabling ejaculatory function.

Large longitudinal SARS-SDAF cohort studies have shown the severe consequences of deafferentation upon sexual function in males – in particular abolishing reflexogenic erection. Here, stimulation of the S2-S4 anterior roots is used to drive erection, though these were often not sufficient for intercourse (Zaer et al., 2018). In the context of female sexual function, findings from one longitudinal study of 130 women, shows no significant changes in sexual function, including genital swelling, orgasm function and ability to engage in sexual intercourse (*p* > 0.05) (Zaer et al., 2018).

Despite sexual function being a high priority for the SCI population (Pettigrew et al., 2021), evidence remains sparse, especially for females. Future studies should study both arousal and orgasm function, validated patient-reported outcome measures (PROMs) such as the International Index of Erectile Function (IIEF) (Rosen et al., 1997) for men and FSFI/FSDS for women, to better delineate how SCS can be safely leveraged to restore sexual function after SCI.

### 4.5 Towards a mechanistic understanding

Mechanistically, SCS is thought to act primarily through recruitment of large-diameter proprioceptive afferents within the dorsal root, which in turn modulate spinal circuitry. In the context of autonomic function, this may translate to the modulation of somato-autonomic reflexes (Samejima et al., 2023b). Whilst the post-ganglionic autonomic neurons are the ultimate effectors, our understanding of how SCS modulates the spinal networks remains limited. Notably, SCS appears to influence these networks in an integrated manner, producing both task-specific excitatory and inhibitory effects, facilitating synergistic improvements across multiple systems. It may be the case that instead of simply driving global sympathetic or parasympathetic activation, SCS may influence the excitability of spinal networks (West et al., 2018), allowing integration of any residual functional networks, rebalancing autonomic tone—though the precise mechanistic basis remains uncertain.

As observed in motor applications, chronic stimulation may also promote adaptive plasticity, improving function, reducing symptom burden and reliance on SCS (Wagner et al., 2018). Greater mechanistic evidence, through preclinical models, uncovering key circuits involved in network control, will be essential in allowing targeted control of autonomic function in SCI.

### 4.6 Neuromodulation strategies

A major neuromodulation strategy, derived from motor studies, has aimed to apply subthreshold tonic stimulation to optimise spinal network excitability (Harkema et al., 2018a; Herrity et al., 2022; Angeli et al., 2024). This has been theorised to allow integration of residual descending supraspinal input, alongside appropriate peripheral sensory feedback, allowing functional networks to emerge (Angeli et al., 2024). Using tonic subthreshold stimulation, is particularly useful as multiple task-specific configurations can be interleaved to allow multi-modal benefits (Angeli et al., 2024). Recently, one study has also applied an alternative stimulation strategy, through patterned spatio-temporal stimulation targeting specific known neuron pools thereby activating sympathetic/parasympathetic preganglionic neurons (Squair et al., 2021).

Transcutaneous SCS also parallels the broad range of recovery achieved with eSCS, and presents a compelling non-invasive alternative to eSCS, carrying fewer risks and cost. Mechanistically, tSCS has demonstrated the ability to activate similar neural substrates to eSCS (Hofstoetter et al., 2018). The evidence base for effective tSCS however, remains smaller than that for eSCS. Crucially, even with mapping, tSCS lacks the spatial specificity offered by eSCS, leading to reduced precision and ability to generate highly task-specific stimulation to maximise functional benefits and minimise off-target effects. Interestingly, dysfunctional responses to stimulation resembling low-grade AD have been reported predominantly in tSCS studies, but not in eSCS (Solinsky et al., 2024; Engel-Haber et al., 2024). Although data remains limited, this suggests that despite targeting similar neural structures, the lower spatial selectivity of tSCS may engage autonomic circuits differently, leading to altered autonomic outputs. The selectivity offered by eSCS remain critical for personalising neuromodulatory strategies, however, must be balanced with the surgical risk and suitability of patients for an eSCS implant.

Historically, Sacral Anterior Root Stimulation has also been utilised for improving bladder function in those with SCI. Our review demonstrates that the SARS-SDAF can reliably modulate pelvic organ function, primarily yielding significant improvements in bladder and bowel function. Although early SARS systems were implanted without deafferentation, SDAF became integral to the procedure, eliminating afferent signals driving events like NDO and AD. The major drawback, however, remains the requirement for irreversible deafferentation, particularly detrimental to erectile and sexual function. Over the years, the use of SARS-SDAF has declined due to its complication rates, highly invasive and irreversible nature, alongside the development of other alternative therapies, namely botulinum toxin and newer neuromodulatory strategies such as SNM, DGNS and SCS (Guiho et al., 2022). Within our review, only two studies evaluated SARS without deafferentation, demonstrating modest improvements in storage but potential worsening of voiding function in the presence of detrusor–sphincter dyssynergia (Arnold et al., 1986; Kirkham et al., 2002). Nonetheless, although recent work has focused predominantly on dorsal cord and root stimulation, we propose that alternative stimulation paradigms should be further explored. Variation of lead and array positions will help fully characterise the therapeutic potential of SCS across different autonomic functions.

### 4.7 Multi-system approaches

In the broader context of SCI, it is essential to view motor and autonomic dysfunction within an interconnected framework. Neural control of motor and autonomic control is deeply intertwined, sharing overlapping circuits, specifically within the lumbosacral cord. Interestingly, one study has showed that isolated locomotor rehabilitation training in patients with both incomplete and complete SCI can also lead to improvements in bladder, bowel and sexual outcomes (Hubscher et al., 2018). Bladder and colonic distension are amongst the most common triggers of AD, whilst haemodynamic instability itself can also limit bladder capacity (Hubscher et al., 2018). Targeted eSCS has been shown to reduce detrusor overactivity and intravesical pressures, mitigating AD induced by bladder distention, whilst enhancing storage capacity (Herrity et al., 2022). Interdependence of pelvic organ function, i.e., bladder-bowel crosstalk has also long been evident (Sinha et al., 2022). Furthermore, beyond direct genital site effects, improvements in continence, bowel function, and mobility may reduce sexual distress and improve body confidence, both of which strongly influence libido, arousal, and sexual satisfaction (Anderson et al., 2007). This psychosocial dimension should not be underestimated in rehabilitation outcomes.

Together these findings highlight the importance of integrated approaches, whereby modulation of systems in parallel, may offer more durable and clinically relevant outcomes for individuals with SCI.

### 4.8 Stimulation parameters and future systems

A survey of US physicians, completed in 2019, highlighted that 83.3% of physicians reported that lack of clear guidelines on stimulation parameters acts as a barrier to wider implantation of SCS (Solinsky et al., 2020). With a poor consensus on the optimal parameters, there is a need for systematic comparison of parameters for autonomic function. Stimulation parameters are task- and individual-specific, making systematic mapping an indispensable step for effective application of SCS – however only 11 out of 30 eSCS studies directly employed mapping procedures to target autonomic function. Mapping for eSCS has been notably fundamental in successful autonomic improvements, within our review. Omission of spatiotemporal and functional mapping is likely to result in reduced efficacy, disconnect between research protocols and clinical benefit, and potentially raise safety concerns (Beck et al., 2021; Solinsky et al., 2024).

In our review, we collated the electrode configurations reported to date (Supplementary Table S2), in only 14 of 30 eSCS studies. More consistent reporting may allow the creation of large configuration libraries, to guide mapping in future studies. Incorporating computer modelling and machine learning algorithms (Mesbah et al., 2019; Zhao et al., 2021) may also help reduce the practical burden of mapping. Ultimately, the challenge will be balancing these optimisation processes with the realities of clinical practise, where time and resources are limited.

To date, most systems implanted were designed for chronic pain applications. In the future, purpose-built electrode arrays should be guided by understanding the neural architecture and anatomy. Wider and longer electrode arrays may allow a more complete coverage of the thoracolumbar and lumbosacral cord with higher specificity.

### 4.9 Limitations

Notably, detail of reporting within studies has been poor. This includes participants characteristics (injury level, grade, and chronicity); stimulation protocols (device type, frequency, intensity, pulse width, waveform, electrode configurations); and intervention duration. Incomplete reporting of outcome measures, e.g., mean and standard deviation of parameters, both before and after the intervention, further precluded quantitative synthesis or meta-analysis, to ascertain any clear effects of SCS. Future studies should aim to follow minimum reporting standards outlined in the Report-SCS guidelines (Malik et al., 2024) to allow reproducible protocols and comparability across studies.

We also note that systematic evaluation of SCS on autonomic thermoregulatory and respiratory dysfunction is lacking. Existing evidence is anecdotal or largely motor focused, with autonomic contributions unexplored. Future work should incorporate comprehensive assessments of thermoregulatory and respiratory responses to clarify these potential effects.

The literature is predominantly comprised of low-level evidence, limited to case series, case reports and few cohort studies. Consequently, the overall certainty of evidence is constrained by inherent risks of bias. Participant cohorts were often highly selective with limited clarity around recruitment strategies. Only one study explicitly reported consecutive recruitment of eligible participants (Samejima et al., 2023a), whilst others presented data from selected subsets or preliminary findings of larger ongoing studies, raising concerns regarding selection bias. Furthermore, most studies only report within-subjects effects of SCS-intervention, with only one study directly comparing effect of SCS-intervention to SCI-controls (Herrity et al., 2021). These limitations were most pronounced for sexual and bowel outcomes, where both the number of studies and sample sizes were smallest.

Accordingly, findings should be interpreted with caution and cannot currently be considered definitive evidence of efficacy of SCS in improving autonomic outcomes in SCI. In contrast, the evidence base for SARS/SDAF is more established, spanning several decades, with recent large retrospective cohort studies supporting its long-term efficacy and safety.

## Data Availability

The original contributions presented in the study are included in the article/Supplementary material, further inquiries can be directed to the corresponding author.

## Footnotes

1
https://www.crd.york.ac.uk/PROSPERO/view/CRD420250621447
