# Supplementary material for: Spinal cord stimulation to manage autonomic dysfunction after spinal cord injury: a systematic review
Source: Front Hum Neurosci. 2026 Jun 3;20:1763475. doi: 10.3389/fnhum.2026.1763475 (PMC13272453; doi:10.3389/fnhum.2026.1763475)
Supplement: Supplementary file 1 [file Table_1.docx]

**Supplementary Table 1**. Stimulation Parameters including type of stimulation (stim) given (epidural (eSCS) or transcutaneous (tSCS) spinal cord stimulation or sacral anterior root stimulation with sacral deafferentation (SARS-SDAF) or without (SARS)), device used, electrode placement and stimulation parameters (waveform, pulsewidth (PW), frequency and intensity). MT=Motor Threshold; NR=Not Reported.

|  |  |  | |  |  |  | | |  | |  | |  |  |
| --- | --- | --- | --- | --- | --- | --- | --- | --- | --- | --- | --- | --- | --- | --- |
| Article | | **Stim type** | **Device** | | | | **Electrode Placement** | | |  | | **Stim Parameters** | | |
|  |  |  |  |  |  |  | **Cathode** | **Anode** | | **Waveform/PW** | | **Frequency** | | **Intensity** |
| Engel-Haber et al. 2024 | | tSCS | NeoStim-5 Constant Current Stimulator, Cosyma, Moscow, Russia | | | | Multi-site C3/4 -- 1/2 | Iliac Crests | | kHz burst/  1 ms (40 μC) | | 30Hz (5kHz) | | NR |
| Solinsky et al. 2024 | | tSCS | Biphasic constant current stimulator (DS8R, Digitimer) | | | | T10-T11 | Iliac Crests | | Biphasic/ kHz burst/1ms | | 30Hz, 120Hz, 30Hz (5kHz); | | 80% MT |
| Hodgkiss et al. 2024 | | eSCS (n = 2) | Restore-ADVANCED, Specify 5-6-5, Medtronic, | | | | T10-12 | | | 380-500μs | | 30-300Hz | | 3.6 - 6.1mA |
|  |  | tSCS (n = 2) | TESCoN, SpineX Inc., California, US) | | | | T11-L1  Vertebral | Iliac crests | | kHz burst  1ms | | 30Hz (10kHz) | | 39-78mA |
| Kumru et al. 2023 | | tSCS | BioStim-5 (Cosyma Inc., Moscow, Russia) | | | | C3-C4 + T9-T10 | Iliac crests | | kHz burst/  1ms; | | 30Hz (10kHz) | | 90% MT |
| Shackleton et al. 2023 | | eSCS | Tripole and Proclaim Elite, Abbott | | | | T11-T12 vertebral | | | NR | | NR | | NR |
| Samejima et al. 2023 | | eSCS | Restore-ADVANCED, Specify 5-6-5 paddle array, Medtronic | | | | T10-T12 vertebral | | | 300-500 μs | | 17 - 35 Hz | | 4-6.8 V |
| Rybka et al. 2023 | | eSCS | WaveWriter alpha IpG, Coveredge X32 surgical leads Boston Scientific | | | | L1-S1 spinal | | | 210 μs | | 20-70 Hz | | 1.8-6.1mA (subthreshold) |
| Gorgey et al. 2023 | | eSCS | Two 8-electrode percutaneous Intellis Medtronic | | | | T10-T12 vertebral | | | 250–1000 μs | | 2-40 Hz | | 1–10 mA |
| Kreydin et al. 2022 | | tSCS | SCONE SpineX Inc, 3 transcutaenous | | | | T11-T12 + L1-L2; | Iliac crests | | Biphasic/ kHz burst/1ms | | 30Hz (10kHz) | | 80% MT |
| Kandhari et al. 2022 | | eSCS | RestoreUltra SureScan, 5-6-5 paddle leads Medtronic | | | | T11-L1 vertebral | | | 270 μs | | 60 Hz | | 1-1.5V |
| Kandhari et al. 2022 | | eSCS | Octad standard lead, LZ STD EAME, Medtronic, | | | | T1-T5 spinal |  | | 210 - 300 µs | | 20-100 Hz | | 2-6 V |
| Samejima et al. 2022 | | tSCS | ONWARD Medical Inc, | | | | Multi-site C3-C4 -- L1 | iliac crests | | kHz burst/  1ms | | 30Hz (10kHz) | | 35-75mA (T11,L1), 5-40mA (C3-C7) |
| Herrity et al. 2022 | | eSCS | Restore-ADVANCED or Intellis, Specify 5-6-5 paddle array, Medtronic | | | | T11-L1 vertebral | | | 450 μs | | 20-85 Hz | | 1.8-12.0mA |
| Sachdeva et al. 2021 | | tSCS | Digitimer DS5, Digitimer Ltd, UK | | | | T7/8 | Iliac Crests | | Biphasic/  2ms | | 30 Hz | | 20–30 mA |
| Squair et al. 2021 | | eSCS | RestoreAdvanced SureScan, 5-6-5 paddle array, Medtronic | | | | T10-11 vertebral | | | NR | | 120 Hz | | NR |
| Herrity et al. 2021 | | eSCS | Restore-ADVANCED, Specify 5-6-5 paddle array, Medtronic | | | | T11-L1 vertebral | | | NR | | NR | | NR |
| DiMarco et al. 2021 | | eSCS | Finetech Medical Ltd., Cough Stimulation System, 2 Percutaneous lead 2 contacts/lead | | | | T9 -- T11 spinal | | | 200 μs | | 50 Hz | | 20-30 V |
| Legg Ditterline et al. 2021 | | eSCS | Restore-ADVANCED, Specify 5-6-5 paddle array, Medtronic | | | | L1-S1 spinal | | | 450 μs | | 30-60 Hz | | NR |
| Beck et al. 2021 | | eSCS | Specify 5-6-5, Medtronic, Fridley, MN | | | | T12-L1 vertebral | | | NR | | NR | | NR |
| Gad et al. 2020 | | tSCS | TESCoN device (spineX, Inc) | | | | C3-4, C5-6, or T1–2; | bilateral shoulders | | kHz burst/ 1 ms; | | 30 Hz (10kHz) | | 20mA |
| DiMarco et al. 2020 | | eSCS | Finetech Medical Ltd., Cough Stimulation System, 2 Percutaenous lead 2 contacts/lead | | | | T9 -- T11 spinal | | | 200 μs | | 50 Hz | | 30-40 V |
| Legg Ditterline et al. 2020 | | eSCS | Restore-ADVANCED, Specify 5-6-5 paddle array, Medtronic | | | | L1-S1 spinal | | | 450 μs | | 30-60 Hz | | NR |
| Nightingale et al. 2019 | | eSCS | Restore-ADVANCED, Specify 5-6-5 paddle array, Medtronic | | | | T11-L1 vertebral | | | 300 μs | | 35 Hz | | 3.5-6.0 V |
| DiMarco et al. 2019 | | eSCS | Finetech Medical Ltd., Cough Stimulation System, 2 Percutaenous lead 2 contacts/lead | | | | T9 -- T11 spinal | | | NR | | NR | | NR |
| Doherty et al. 2019 | | tSCS | Digitimer DS7, Digitimer Ltd., United Kingdom) | | | | T11-12 | abdominal areas | | 200 μs | | 15 Hz | | NR |
| Darrow et al. 2019 | | eSCS | Primary cell IPG Proclaim Elite™, Abbott, 16 contact paddle (5-6-5) | | | | T12 Vertebral | | | 200 - 500 μS | | 16 - 400 Hz | | 2 -15mA |
| Phillips et al. 2018 | | tSCS | NR | | | | T8 spinal segment | Iliac Crest | | 1ms | | 30 Hz | | 70mA |
| Herrity et al. 2018 | | eSCS | RestoreADVANCED, 5-6-5 paddle array, Medtronic | | | | NR | | | 450 μS | | 5 - 60 Hz | | 0.5-3V |
| Walter et al. 2018 | | eSCS | RestoreADVANCED, 5-6-5 paddle array, Medtronic | | | | T11-L1 Vertebral | | | 300-450 μs | | 30 - 45 Hz | | 4 - 7 V |
| Gad et al. 2018 | | tSCS | NeuroRecovery Technologies, Inc | | | | T11-T12, and L1-L2; | Iliac Crests | | 1ms | | 30 Hz | | 10–200 mA |
| Zaer et al. 2018 | | SARS-SDAF | Finetech-Brindley, percutaneous lead electrodes | | | | intrathecal S2-S4 anterior roots | | | Intermittent | | NR | | NR |
| DiMarco et al. 2018 | | eSCS | NR (2 single electrode leads) | | | | T9 -- T11 spinal | | | 200 μs | | 50 Hz | | 40 V |
| Harkema et al. 2018(b) | | eSCS | Restore-ADVANCED, Specify 5-6-5 paddle array, Medtronic | | | | L1-S1 spinal segments | | | 450 μs | | 30-60 Hz | | NR |
| Aslan et al. 2018 | | eSCS | Restore-ADVANCED, Specify 5-6-5 paddle array, Medtronic | | | | L1-S1 spinal segments | | | NR | | 15-30 Hz | | 2.4 - 7.5 V |
| Harkema et al. 2018 (a) | | eSCS | Restore-ADVANCED neurostimulator, Specify 5-6-5 paddle array (16-electrode array), Medtronic | | | | L1-S1 spinal segments | | | 450 μs | | 30-60 Hz | | NR |
| West et al. 2018 | | eSCS | Restore-ADVANCED neurostimulator, Specify 5-6-5 paddle array (16-electrode array), Medtronic | | | | T11-L1 vertebral | | | 300ms | | 35 Hz | | 3.5 V |
| Krebs et al. 2017 | | SARS-SDAF | Brindley stimulator | | | | S2-S5 anterior roots | | | Intermittent | | NR | | NR |
| Castano-Botero et al. 2016 | | SARS-SDAF | Finetech-Brindley, percutaneous lead electrodes | | | | Extradural S2-S4 anterior roots | | | Intermittent | | NR | | NR |
| Rasmussen et al. 2015 | | SARS-SDAF | Brindley stimulator | | | | S2-S4 anterior roots | | | Intermittent | | NR | | NR |
| Krasmik et al. 2014 | | SARS-SDAF | Brindley stimulator | | | | S2-S4 anterior roots | | | Intermittent | | NR | | NR |
| DiMarco et al. 2014 | | eSCS | Freehand Epimysial Electrode; NeuroControl Corp., 8333 Rockside Road, Valley View, OH | | | | T9, T11 and L1 spinal | | | Intermittent | | NR | | NR |
| DiMarco et al. 2009 | | eSCS | Freehand Epimysial Electrode; NeuroControl Corp, 8333 Rockside Rd, Valley View, OH 44125. | | | | T9, T11 and L1 spinal | | | 200 μs  Intermittent | | 50 Hz | | 10-40 V |
| Valles et al. 2009 | | SARS-SDAF | Finetech-Brindley Bladder system or Vocare | | | | S2-S4 anterior roots | | | Intermittent | | NR | | NR |
| DiMarco et al. 2006 | | eSCS | Freehand Epimysial Electrode; NeuroControl Corp, 8333 Rockside Rd, Valley View, OH 44125." | | | | T9, T11 and L1 spinal | | | 150 - 200 μs  Intermittent | | 53 Hz | | 40 V |
| DiMarco et al. 2005 | | eSCS | Atrotecha tripolar epidural disk electrode (TF 3-3-U) or Medtronicb quadripolar spinal cord electrode | | | | T2-3 vertebral | | | 200 μs  Intermittent | | 8.6-28Hz | | 0-38 V |
| Kirkham et al. 2002 | | SARS | Finetech-Brindley, percutaneous lead electrodes | | | | S2-S4 mixed roots | | | 8 - 256 μs  Intermittent (voiding) | | 15 Hz | | NR |
| van der Aa et al. 1999 | | SARS-SDAF | Brindley bladder stimulator implant | | | | S2-S5 anterior roots | | | Intermittent | | NR | | NR |
| Egon et al. 1998 | | SARS-SDAF | Finetech-Brindley, percutaneous lead electrodes | | | | S2-S4 anterior roots; | | | Intermittent | | NR | | NR |
| Schurch et al. 1997 | | SARS-SDAF | NR | | | | S2-S4 anterior roots | | | Intermittent | | NR | | NR |
| Van Kerrebroeck et al. 1997 | | SARS-SDAF | Finetech-Brindley | | | | S2-S4 anterior roots | | | Intermittent | | NR | | NR |
| Van Kerrebroeck et al. 1996 | | SARS-SDAF | Finetech-Brindley | | | | S2-S4 anterior roots | | | Intermittent | | NR | | NR |
| Van der Aa et al. 1995 | | SARS-SDAF | Finetech-Brindley, percutaneous lead electrodes | | | | S2-S5 anterior roots | | | Intermittent | | NR | | NR |
| Koldewijn et al. 1994 | | SARS-SDAF | Brindley-Finetech sacral anterior root stimulator | | | | S2-S5 anterior roots | | | Intermittent | | NR | | NR |
| Katz et al. 1991 | | eSCS | Medtronic Inc., Resume lead model 7492) | | | | T1 vertebral tetra; T11-12 para | | | 180-450ms | | 25-130 Hz | | 0.25-10.5 V |
| MacDonagh et al. 1990 | | SARS-SDAF | Brindley-Finetech sacral anterior root stimulator | | | | S2-S4 anterior roots | | | 100-600μs  Intermittent | | 10-20Hz | | 10-40 V |
| Arnold et al. 1986 | | SARS | Brindley-Finetech Sacral Anterior Root Stimulator | | | | S2-S4 anterior roots | | | Intermittent | | NR | | NR |
