# Supplementary material for: Spinal cord stimulation to manage autonomic dysfunction after spinal cord injury: a systematic review
Source: Front Hum Neurosci. 2026 Jun 3;20:1763475. doi: 10.3389/fnhum.2026.1763475 (PMC13272453; doi:10.3389/fnhum.2026.1763475)
Supplement: Supplementary file 2 [file Data_Sheet_1.docx]

**Supplementary Table 2**: eSCS devices, level of implantation, anode/cathode array configurations and stimulation parameters (frequency, pulse width) used across the included studies by autonomic function. Optimal configurations are those identified post-mapping. Other configurations that were tested, have been documented within the table by autonomic function, where no specific mapping was undertaken. Anodes (+, red), Cathodes (-, blue); Inactive Electrodes (grey).

**Cardiovascular Function**

| **Study** | **Device Implantation** | **Optimal Configuration** | **Other Tested Configurations** |
| --- | --- | --- | --- |
| **Squair et al. 2021** | RestoreAdvanced SureScan neurostimulator,  5-6-5 paddle array (16-electrode array), Medtronic  Vertebral Level:  T10 – 11 | 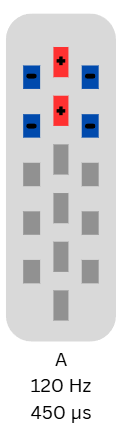 | 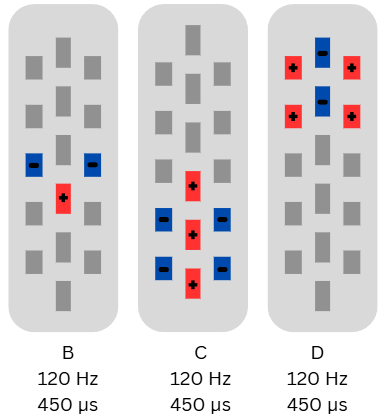 |
|  | Comment | (A) Activation of the rostral part of the array to target the caudal thoracic cord (T10-12 spinal segments) allows a controlled SBP rise of 39.9 ± 3.4 mmHg. Cathodes (-) are placed laterally to preferentially target the dorsal roots | (B) Activation of the middle part of the array and the (C) caudal part of the array leads to a reduced rise in SBP of 13.8 ± 8.4 and 4.5 ± 3.8 respectively. (D) Placing the Cathodes (-) in the midline compared to lateral position preferentially targets the posterior column rather than posterior roots. This led to little or no rise in SBP. |
| **Harkema et al. 2018 + Legg-Ditterline et al. 2021** | RestoreADVANCED neurostimulator,  5-6-5 paddle array (16-electrode array), Medtronic  Vertebral Level:  T11-L1 | 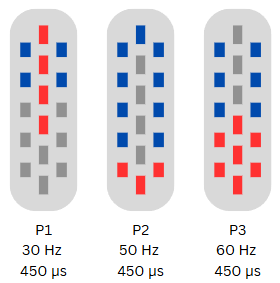  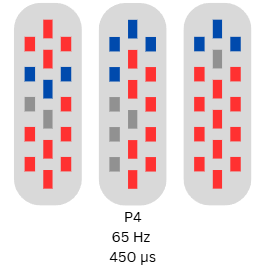 | 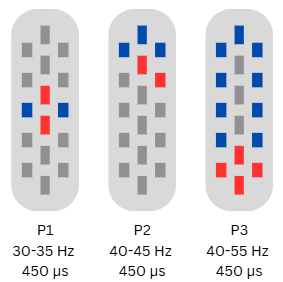  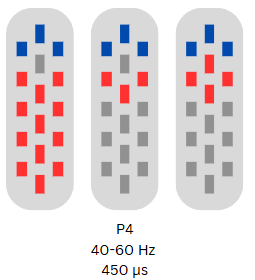 |
|  | Comment | The optimal configurations of 4 patients that can maintain the SBP in a normative range of 110-120mmHg. | These are configurations of the same 4 patients – that were unable to maintain the SBP in a normative range (110-120mmHg) and even dropped below 90mmHg in some cases.  In both optimal and non-optimal configurations, cathodes have been placed in a rostral position, to try and direct the field towards the caudal thoracic cord. Cathode-Anode distribution pattern can hence drastically impact the electric field and its ability to target the desired spinal levels. |
| **Hodgkiss et al. 2024** | RestoreADVANCED neurostimulator,  5-6-5 paddle array (16-electrode array), Medtronic  Vertebral Level:  T10-12 or T11-L1 | 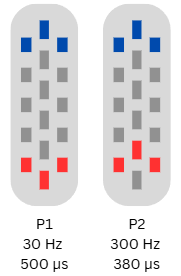 | 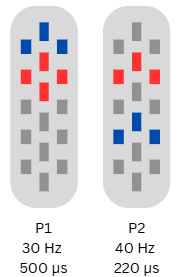 |
|  | Comment | Stimulation led to a rise in resting SBP of +12 (104 to 116mmHg) in P1; and of +11 (134 to 145mmHg) in P2. Both P1 and P2 seemed to benefit where cathodes were placed rostrally leading to caudal stimulation of the thoracic cord. | Sham-stimulation configuration led to change in resting of SBP of -1 (100 to 99mmHg) in P1; and of -2 (129 to 127 mmHg) in P2. In P2 where cathodes were places in a more caudal position stimulation was no longer effective at increasing SBP |
| **Darrow et al. 2019** | Primary cell IPG Proclaim Elite™, Abbott, 16 contact array (5-6-5)  Vertebral Level:  T12 | 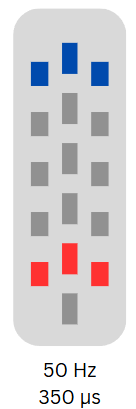 | 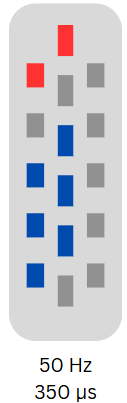 |
|  | Comment | Stimulation in the rostral direction ameliorated SBP drop during a tilt-table test from -34.33 to +6.83 mmHg (estimated using software) leading to recovery of BP and resolution of any symptoms of OH | Sham-stimulation applied in the caudal direction was unable to maintain BP during tilt-table test or improve any symptoms of OH. |
| **West et al. 2018 + Nightingale et al. 2019** | RestoreADVANCED neurostimulator,  5-6-5 paddle array (16-electrode array), Medtronic  Vertebral Level:  T11-L1 | 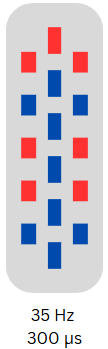 | NR |
|  | Comment | The above configuration was shown to modulate CVS function in the same individual studied by both West et al. 2018 and Nightingale et al. 2019. Stimulation led to an amelioration in SBP drop from -30 to +3 mmHg during a Tilt-Table test. Stimulation also increased resting MAP by +14mmHg in the same individual – in this case increasing stimulation intensity had no further effect on increase BP. | N/A |
| **Study** | **Device** |  |  |
| **Samejima et al. 2023** | RestoreADVANCED neurostimulator,  5-6-5 paddle array (16-electrode array), Medtronic  Vertebral Level:  T10-T12 | NR | 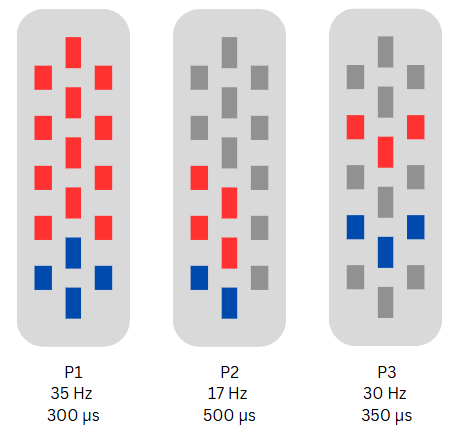 |
|  | Comment | N/A | Cathodes were placed at the caudal parts to target the segments of the caudal lumbosacral cord associated with bowel control.  DARS test was performed to trigger AD in 3 patients – Stimulation suppressed AD response from +31 ± 14 to +16 ± 0.2 in P1; +22 ± 1 to +13 ± 3 in P2; +26 ± 2 to +8 ± 5 in P3. Hence, acute eSCS prevented episodes of AD by reducing elevation of SBP under > 20mmHg threshold. |
| **Herrity et al. 2022** | RestoreADVANCED or Intellis neurostimulator,  5-6-5 paddle array (16-electrode array), Medtronic  Vertebral Level:  T11-L1 | NR | 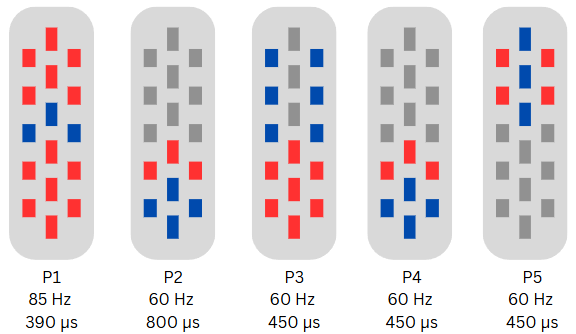 |
|  | Comment | N/A | Mapping was performed to specifically target bladder function in 5 patients with SCI. Configurations targeting the L1-L3/4 spinal levels were found to be optimal for 4/5 patients for improving bladder compliance but also preventing any BP rise in response to bladder stretch (a common trigger for AD). Mean SBP during bladder filling fell by over 36mmHg (157 ± 7 to 121 ± 13 mmHg) showing this configuration was effective at also stabilising SBP. |
| **Aslan et al. 2018** | RestoreADVANCED neurostimulator,  5-6-5 paddle array (16-electrode array), Medtronic  Vertebral Level:  T11-L1 | NR | 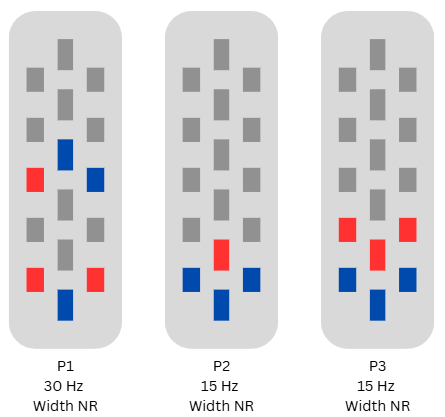  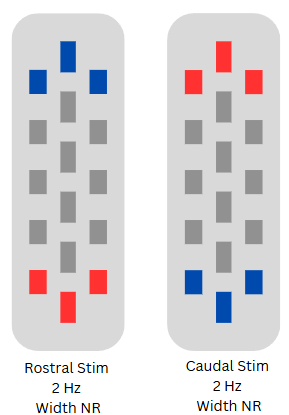 |
|  | Comment | N/A | (a) Mapping was performed in 3 patients with lumbosacral implants to facilitate locomotor function in the lower limbs. The optimal configuration identified mainly can be seen to target caudal parts of the array targeted to activate lower limb musculature. Stimulation led to amelioration in SBP drop (OH) during Sit-Up and Stand Test from -33 to +15.3 mmHg likely due to skeletal muscle pump function.  (b) Further testing showed there was no significant difference in change in SBP when comparing the impact of rostral vs caudal stimulation configurations. 2 Hz was utilised to assess the impact on CVS function. |

**Bladder Function**

| **Study** | **Device** | **Optimal Storage Configuration** | **Optimal Voiding Configuration** | **Other Tested Configurations** |
| --- | --- | --- | --- | --- |
| **Herrity et al. 2022** | RestoreADVANCED or Intellis neurostimulator,  5-6-5 paddle array (16-electrode array), Medtronic  Vertebral Level:  T11-L1 | 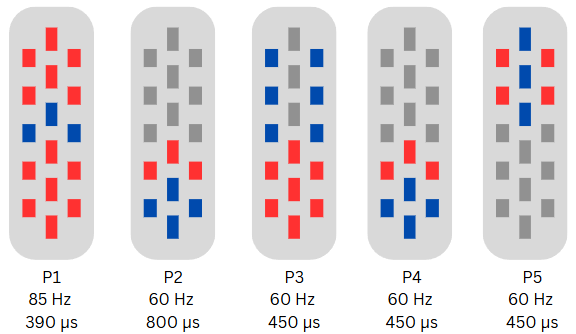 | 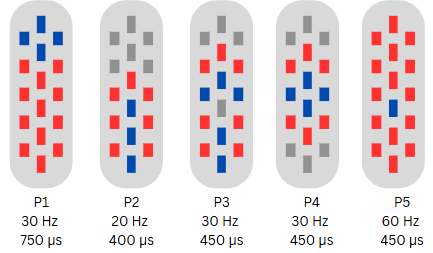 | NR |
|  | Comment | Bladder-specific mapping was conducted in 5 patients to improve bladder compliance for storage function. Optimal Configurations for P1, P3, P4, P5 were those that targeted the rostral to mid lumbosacral cord i.e. L1 - L3/L4. For P2 targeting the caudal sacral region was effective for improving compliance | Bladder-specific mapping was conducted in 5 patients to improve voiding function. Optimal Configurations for P1, P2, P3, P4 for were those that targeted the caudal lumbosacral enlargement i.e. L4 – S. For P5 targeting the L1 segment was effective for voiding. | N/A |
| **Herrity et al. 2018** | RestoreADVANCED neurostimulator,  5-6-5 paddle array (16-electrode array), Medtronic  Vertebral Level:  T11-L1 | NR | 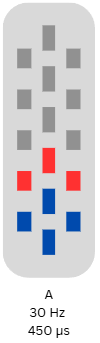 | NR |
|  | Comment | N/A | Bladder-mapping was conducted in an individual to improve voiding function. Optimal configuration (A) was one that targeted the caudal lumbosacral enlargement i.e. L5/S1. This led to a Voiding Efficiency of 88.1 ± 1.1%. Out of all the frequencies tested from 5-60Hz; 30Hz was identified to be the most effective with 45 and 60 Hz also leading to similar voiding efficiencies | N/A |
| **Darrow et al. 2019** | Primary cell IPG Proclaim Elite™, Abbott, 16 contact array (5-6-5)  Vertebral Level:  T12 | NR | NR | 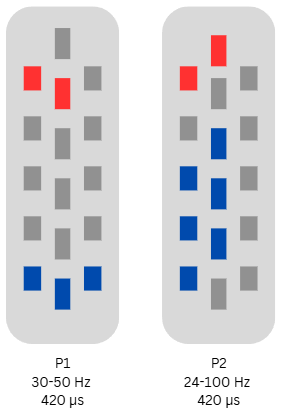 |
|  | Comment | N/A | N/A | The above 2 configuration were designed to target locomotor function i.e. stand/step for 2 individuals (P1 and P2). Over the course of five monthly visits, P1 showed minor improvement in the Storage and Voiding subdomain of the NBSS (9 points to 5 points at the end of the five months). P2 had a significant improvement in the Incontinence subdomain almost immediately (7 points to 0 points) with no improvement or unclear changes in all other subdomain values. |
| **Kandhari et al. 2022** | RestoreULTRA SureScan neurostimulator,  5-6-5 paddle array (16-electrode array), Medtronic  Vertebral Level:  T11-L1 | NR | NR | 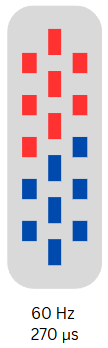 |
|  | Comment | N/A | N/A | 10 participants applied a ‘self-training’ programme at subthreshold stimulation levels for 12-16 hours a day. This was a wide-field configuration using the rostral 8 electrodes as anodes and the caudal 8 as cathodes. Participants reported subjective improvements in sensation of bladder fullness (triggering catheterisation) and fewer incontinence episodes. |

**Bowel Function**

| **Study** | **Device** | **Tested Configurations** |
| --- | --- | --- |
| **Walter et al. 2018** | RestoreAdvanced SureScan neurostimulator,  5-6-5 paddle array (16-electrode array), Medtronic  Vertebral Level:  T11-L1 | 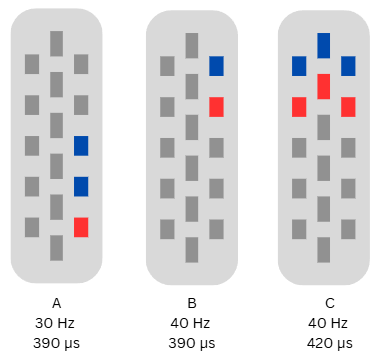 |
|  | Comment | A range of pre-set programme present on the stimulator were tested in a single individual i.e. specific bowel mapping was not conducted. BM times without stimulation at baseline was 58 ± 3 min. When stimulation was applied the above 3 configurations improved BM time to (A) 25 ± 3 min; (B) 23 ± 1 (p < 0.05) (C) 31 ± 4 min. |
| **Samejima et al. 2023** | RestoreADVANCED neurostimulator,  5-6-5 paddle array (16-electrode array), Medtronic  Vertebral Level:  T10-T12 | 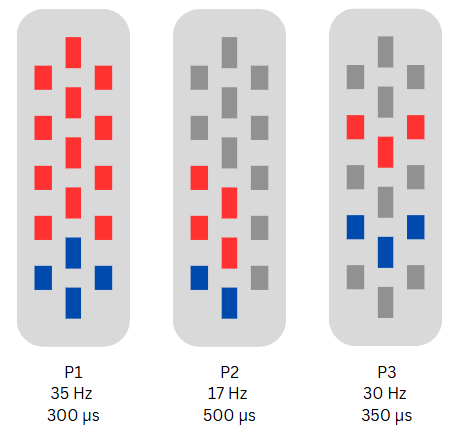 |
|  | Comment | Although parameters related to bowel function were not examined in this study, the above configuration was designed to target bowel function to reduce BP response to DARS. To target bowel function, stimulation mainly targeted caudal areas of the lumbosacral region. |
| **Darrow et al. 2019** | Primary cell IPG Proclaim Elite™, Abbott, 16 contact array (5-6-5)  Vertebral Level:  T12 | 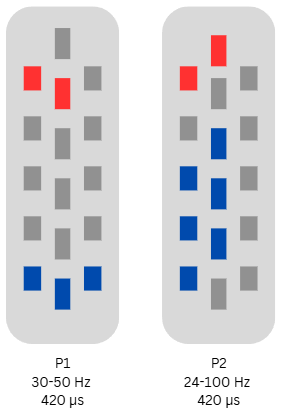 |
|  | Comment | The above 2 configurations were designed to target locomotor function i.e. stand/step for 2 individuals (P1 and P2). Over the course of five monthly visits, P1 showed no change in the NBDS score across the five-month follow-up period. P2 underwent a slight worsening of bowel function reflected by NBDS severity change from Moderate to Severe. However, it is important to note that P2’s bowel regimen time significantly decreased from 90 min to under 30 min. |
| **Kandhari et al. 2022** | RestoreULTRA SureScan neurostimulator,  5-6-5 paddle array (16-electrode array), Medtronic  Vertebral Level:  T11-L1 | 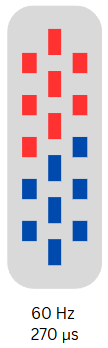 |
|  | Comment | 10 participants applied a ‘self-training’ programme at subthreshold stimulation levels for 12-16 hours a day. This was a wide-field configuration using the rostral 8 electrodes as anodes and the caudal 8 as cathodes. The participant reported a reduction in BM time from 25 mins to 17mins, as well as an increased awareness of bowel fullness. |

**Sexual Function**

| **Study** | **Device** | **Tested Configurations** |
| --- | --- | --- |
| **Rybka et al. 2023** | WaveWriter  Alpha IPG, CoverEdge X32 (32-electrode array) Boston Scientific  Vertebral Level:  T11-L1 | 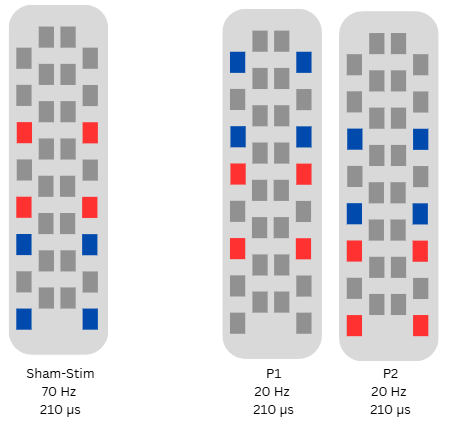 |
|  | Comment | The above configurations were tested in participants to improve ejaculatory function. Targeting the L4 segments by stimulating more rostral rather than caudal regions of the array proved most effective, as demonstrated with configurations (b, c) in two patients. While sham stimulation (a) produced no response, optimized stimulation enabled ejaculation in response to penile vibratory stimulation within 117 seconds in P1 and 72 seconds in P2 |
| **Darrow et al. 2019** | Primary cell IPG Proclaim Elite™, Abbott, 16 contact array (5-6-5)  Vertebral Level:  T12 | 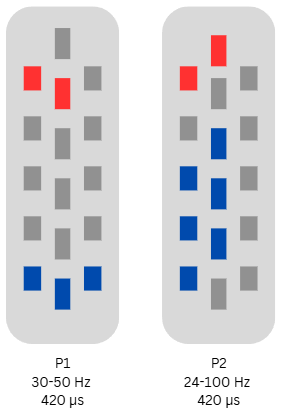 |
|  | Comment | The above 2 configurations were designed to target locomotor function i.e. stand/step for 2 individuals (P1 and P2). Over the course of five monthly visits, P1 reported no change in sexual function. However, P2 reported consistent ability to achieve orgasm with active stimulation – something that was absent since SCI and when stimulation was stopped. |
| **Kandhari et al. 2022** | RestoreULTRA SureScan neurostimulator,  5-6-5 paddle array (16-electrode array), Medtronic  Vertebral Level:  T11-L1 | 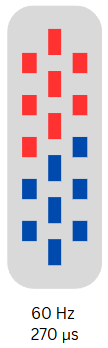 |
|  | Comment | 10 participants applied a ‘self-training’ programme at subthreshold stimulation levels for 12-16 hours a day. This was a wide-field configuration using the rostral 8 electrodes as anodes and the caudal 8 as cathodes. 5/10 participants reported improvements in either reflex or psychogenic erections – however none of them were able to achieve ejaculation. |
